# Supplementary material for: Encoding ordered structural complexity to covalent organic frameworks
Source: Nat Commun. 2024 Mar 18;15:2411. doi: 10.1038/s41467-024-46849-w (PMC10948875; doi:10.1038/s41467-024-46849-w)
Supplement: Supplementary file 1 — Supplementary information [file 41467_2024_46849_MOESM1_ESM.pdf]

Supplementary Information (59 pages)

Encoding Ordered Structural Complexity to Covalent Organic Frameworks

Lei Wei[1,#], Xinyue Hai[1,#], Tongtong Xu[1], Zidi Wang[1], Wentao Jiang[1], Shan Jiang[1], Qisheng Wang[2], Yue-Biao Zhang[1,3]*, Yingbo Zhao[1,3]*

[1]*School of Physical Science and Technology, ShanghaiTech University, Shanghai, 201210, P. R. China.*

[2]*Shanghai Key Laboratory of High-Resolution Electron Microscopy, ShanghaiTech University, Shanghai 201210, China.*

[3]*Shanghai Synchrotron Radiation Facility, Shanghai Advanced Research Institute, Chinese Academic of Sciences, Shanghai 201210, China.*

*To whom correspondence should be addressed: ybzhang@shanghaitech.edu.cn; zhaoyb2@shanghaitech.edu.cn

[#]These authors contributed equally.

Table of contents

Supplementary Note 1. Synthesis of COFs and their structural analogues. 3

Supplementary Note 2. The characterization of the crystalline structures of COF-305 and COF-304... 6

Supplementary Note 3. Structural analysis of COF-304 and COF-305 21

Supplementary Note 4. Structure analysis of model molecules of analogue of COFs............... 36

Supplementary References.. 59

Supplementary Note 1. Synthesis of COFs and their structural analogues.

Synthesis of single-crystalline COF-304. DHPA (15 mg, 0.090 mmol) was dissolved in 0.5 mL of 1,4-dioxane and 60 μL of aniline was added. The mixture was transferred to an NMR tube and then 0.2 mL of aqueous acetic acid (15 M) was added. After homogenously mixed, red-crystalline precipitate was observed, and the solution of TAM (20 mg, 0.0528 mmol) dissolved in 0.75 mL 1,4-dioxane was added carefully. The reaction was placed at room temperature stand still for three weeks, the dark red-coloured crystals slowly crystallized out. The as-synthesized single crystals can easily isolate from the mixture compounds and then exchanged by *n*-butanol three times for SXRD measurement. A rod-shaped crystal ($10 \times 10 \times 60$ μm^3) of COF-304 was selected for SXRD measurement.

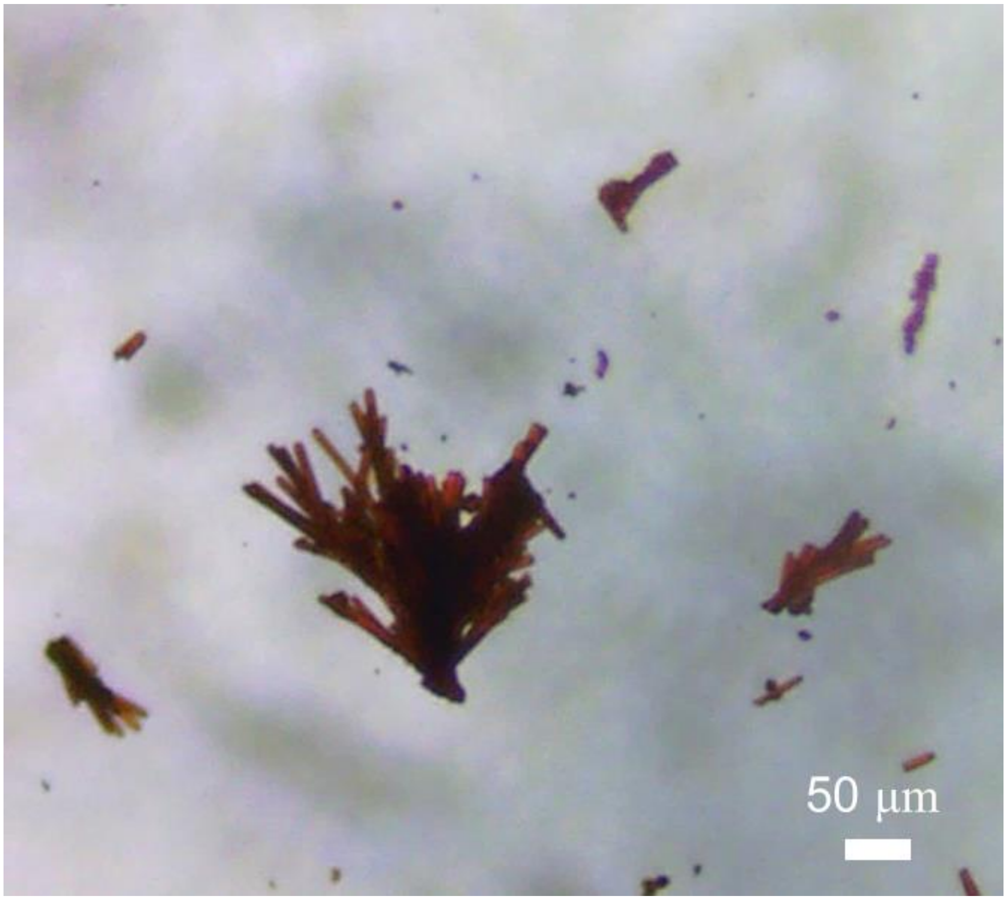

Supplementary Fig. 1. Optical microscopic image of rod-like COF-304.

Synthesis of single-crystalline COF-305. DMPA (17 mg, 0.088 mmol) was dissolved in 0,5 mL of 1,4-dioxane, and then 40 μL of aniline and 200 μL aqueous acetic acid (15M) were added to a 5 mL vial. After homogenously mixing the solution, the solution of TAM (20 mg, 0.0528 mmol) dissolved in 0.75 mL 1,4-dioxane was added carefully. The reaction was placed standstill at room temperature. The yellow-coloured crystals of about 30 μm was crystallized out after two days, and the crystal size could reach about 40 μm in four days. The as-synthesized single crystals were isolated and then exchanged by *n*-butanol for three times for SXRD measurement.

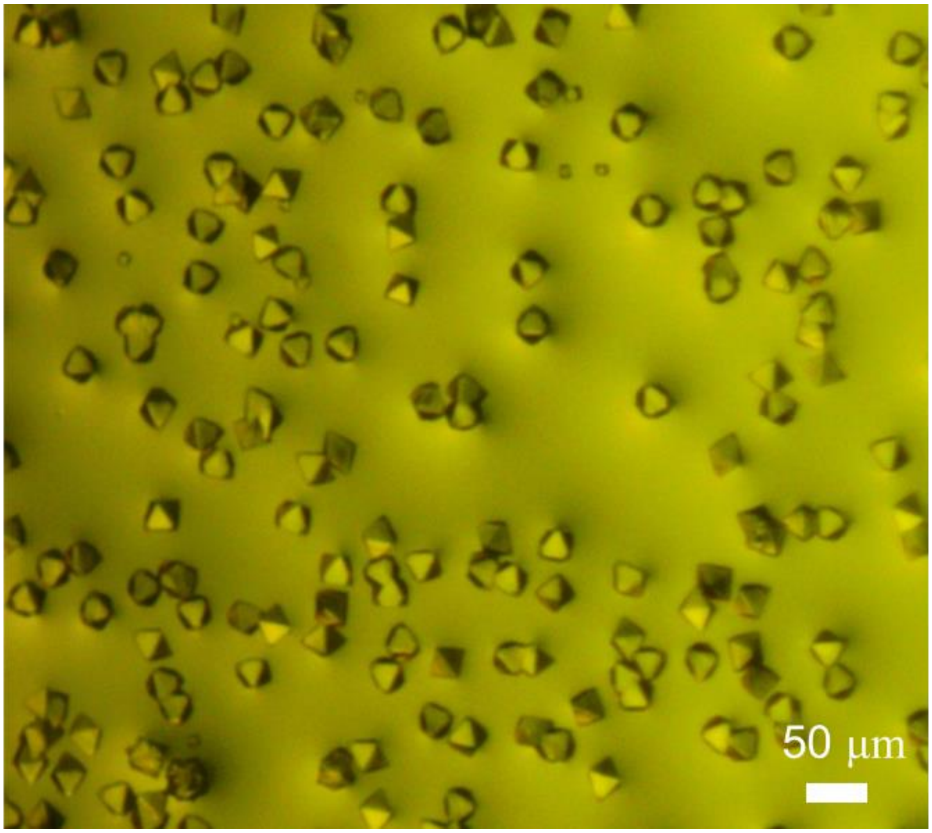

Supplementary Fig. 2. Optical microscopic image of as-synthesized COF-305 crystal with average sizes of 40 μm.

Synthesis of molecular model compound M-300 (1,1'-(1,4-phenylene)bis(N-phenylmethanimine))

TPA (20 mg, 0.149 mmol) was dissolved in 1 mL of 1,4-dioxane, and then 100 μL of aniline and 200 μL aqueous acetic acid (6 M) were added to a 5 mL vial. The reaction was placed standstill at room temperature for half an hour, and a colourless crystal was observed. The as-synthesized single crystals were isolated for SXRD measurement.

Synthesis of molecular model compound M-304 (3,6-bis(-(phenylimino)methyl)benzene-1,2-diol).

DHPA (20 mg, 0.12 mmol) was dissolved in 1 mL of 1,4-dioxane, and then 100 μL of aniline and 200 μL aqueous acetic acid (6 M) were added to a 5 mL vial. The reaction was heated at 80 ^{o}C for one day, then placed at room temperature for one day, and dark red-color crystals were observed. The as-synthesized single crystals were isolated for SXRD measurement.

Synthesis of molecular model compound M-305 (-1,1'-(2,3-dimethoxy-1,4-phenylene)bis(N-phenylmethanimine)).

A mixture of DMPA (23 mg, 0.119 mmol) was dissolved in 0.5 mL of 1,4-dioxane, and then 30 μL of aniline was added to the 5 mL vial. The reaction was placed at room temperature while volatilizing the organic solvent slowly and a yellowish single crystal was obtained. The single crystals were picked up directly for SXRD measurements.

Synthesis of molecular model compound M-305-2 ((1E,1'E,1''E,1'''E)-N,N',N'',N'''-(methanetetrayltetrakis(benzene-4,1-diyl))tetrakis(1-(2,3-dimethoxyphenyl)methanimine)).

A mixture of DMPA (21 mg, 0.119 mmol) was dissolved in 0.5 mL of 1,4-dioxane, and then 10 mg of TAM was added to the 5 mL vial. The reaction was placed at room temperature while volatilizing the organic solvent slowly and a yellowish single crystal was obtained. The single crystals were picked up directly for SXRD measurements.

Supplementary Note 2. The characterization of the crystalline structures of COF-305 and COF-304

Supplementary Table 1. Crystallographic data and structural determination for COF-305.

**Name**	**COF-305**
Formula sum	$C_{1125}H_{906}N_{100}O_{100}$
Formula Weight	17425
Crystal system	Orthorhombic
Space group	*Fdd*2 (No.43)
a (Å)	47.077(9)
b (Å)	67.629(14)
c (Å)	42.547(9)
V (Å^3)	135460 (48)
Z	288
Temperature (K)	100
Density(g/cm^3)	0.615
Measured reflections	38304
unique reflections	38304
Θ range (°)	1.021- 20.841
R_1	0.0744
wR_2	0.2488
S(GOF)	1.004
Parameters	2141
Restraints	190
Max/min res. Dens., (e Å^{-3})	0.220 / -0.171
Crystal size, mm^3	0.06×0.04×0.04
Radiation, Å	0.6887
CCDC number	2292629

$^aR_1 = \sum||Fo| - |Fc||/\sum|F_o|$; $^bwR_2 = [\sum w\,(F_0^2 - F_C^2)^2/\sum w\,(F_0^2)^2]^{1/2}$; $^cS = [\sum w(F_0^2 - F_C^2)^2\,/(N_{ref} - N_{par})]^{1/2}$.

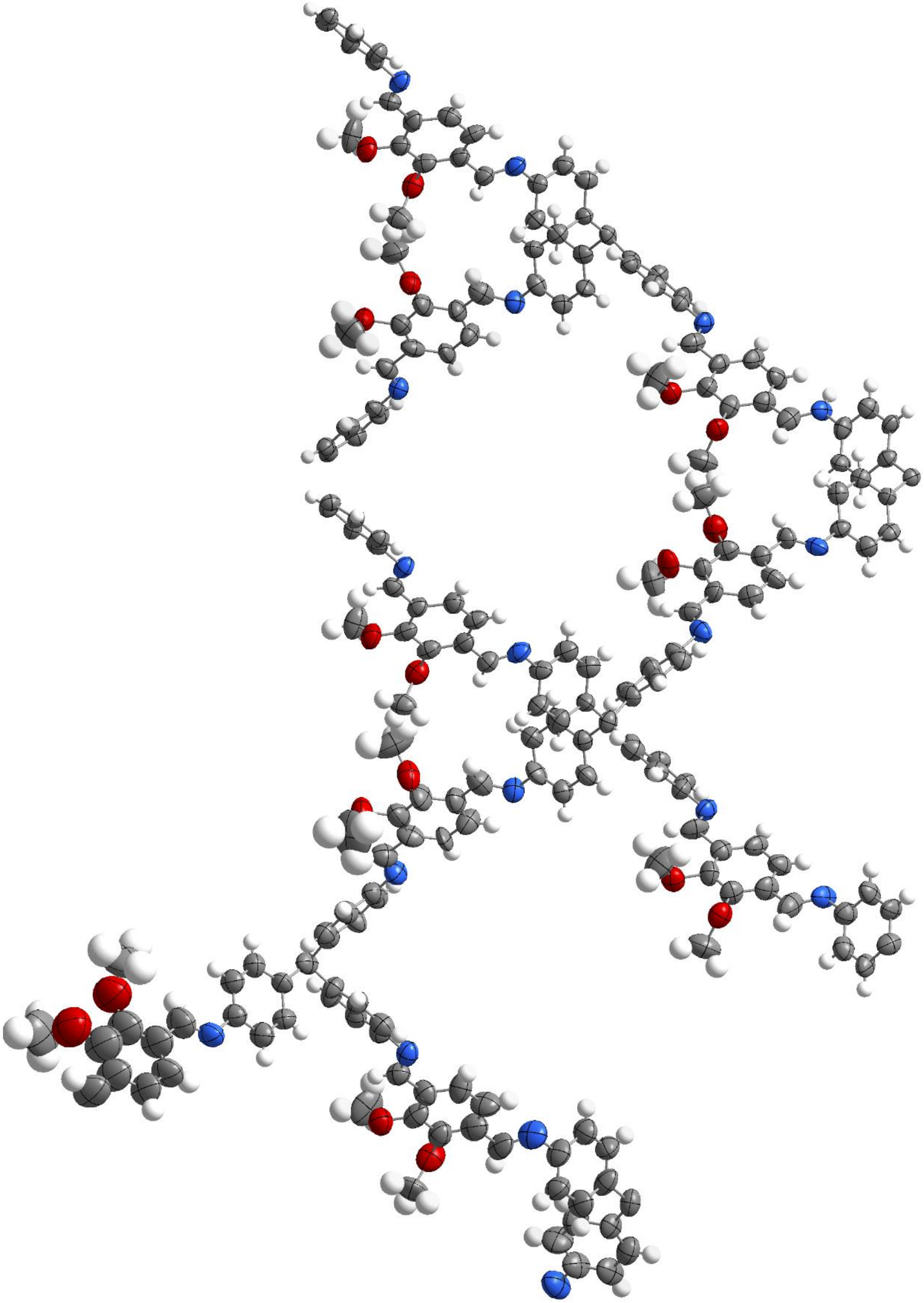

Supplementary Fig. 3. Asymmetric unit in the single-crystal structure of COF-305. Thermal ellipsoids are drawn with a 50% probability. Colour code: N, blue; O, red; C, grey; H, white.

Supplementary Table 2. Unit cell parameters of COF-305 measured under different solvents.

Solvent	Crystal system	*a* (Å)	*b* (Å)	*c* (Å)	*V* (Å^3)
n-butane	*Fdd*2	47.08	67.63	42.55	135460
1,4-dioxane	*Fdd*2	46.52	66.11	41.82	128590
DMF	*Fdd*2	47.23	67.02	42.50	134548
Mesitylene	*Fdd*2	47.35	67.42	42.16	134590

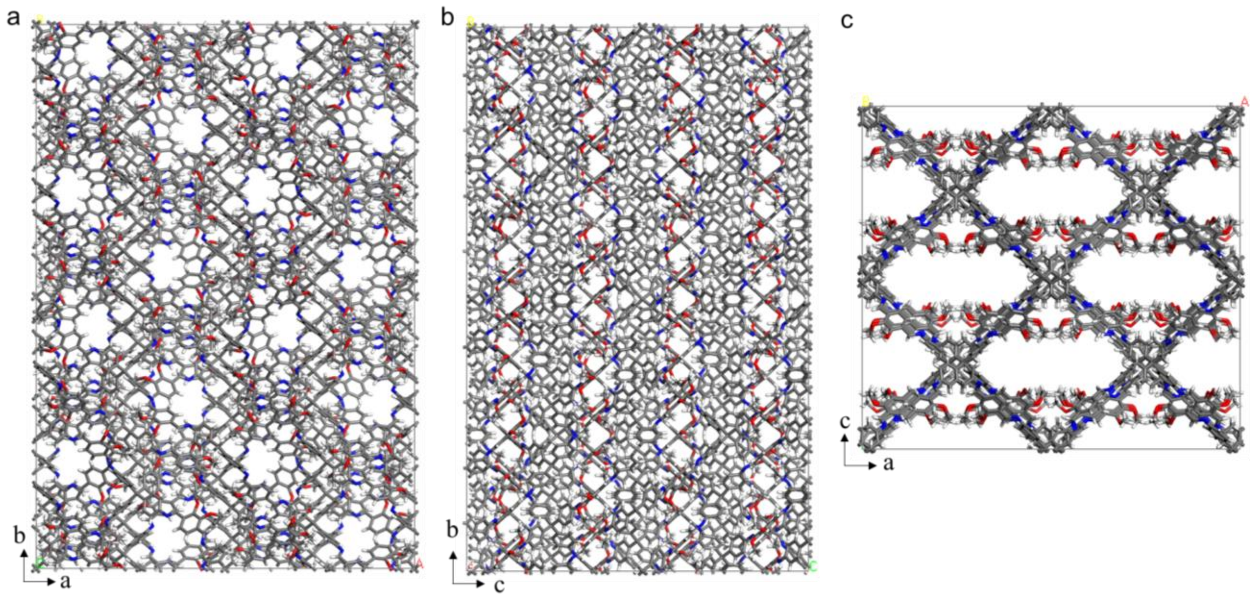

Supplementary Fig. 4. Single-crystal structure of COF-305 viewed from *c*-axis (a), *a*-axis (b), and *b*-axis (c). Colour code: N, blue; O, red; C, grey; H, white.

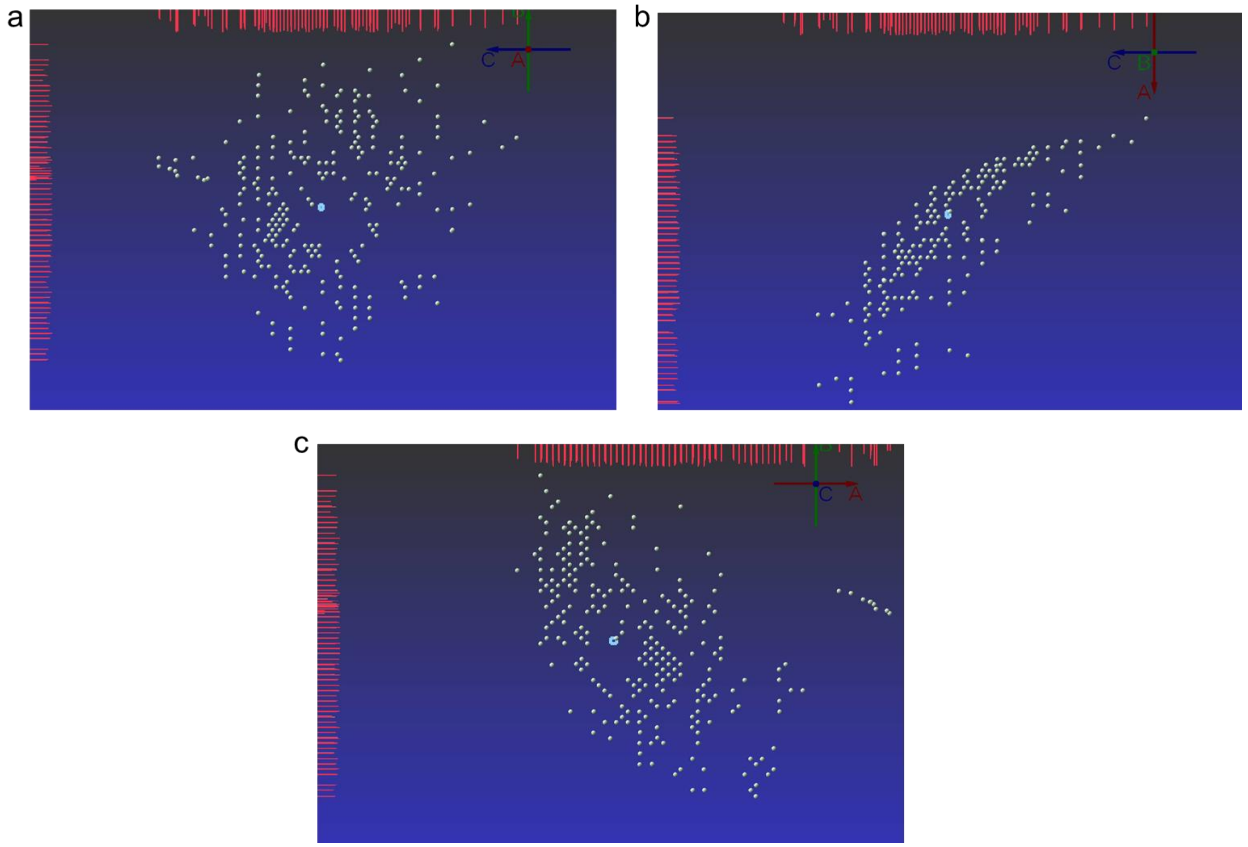

Supplementary Fig. 5. The oriented lattice views. The 2D profiles on the top and the left are counting intensity. Figure a) viewed from the *a*-axis, (b) from the *b*-axis, and (c) from *c*-axis.

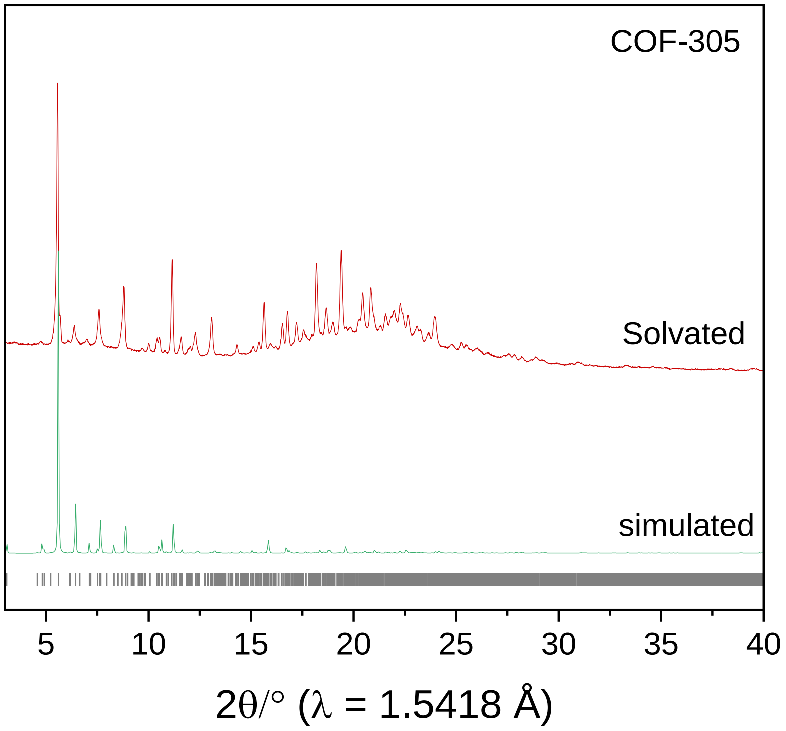

Supplementary Fig. 6. PXRD patterns of COF-305 collected in *n*-butanol vapor atmosphere (red). The experimental pattern is compared to the simulated pattern based on the refined single crystal structure (green). The difference in intensity between experimental pattern and simulated one is due to the calculated PXRD pattern according to the "solvent-masked" structure of COF-305.

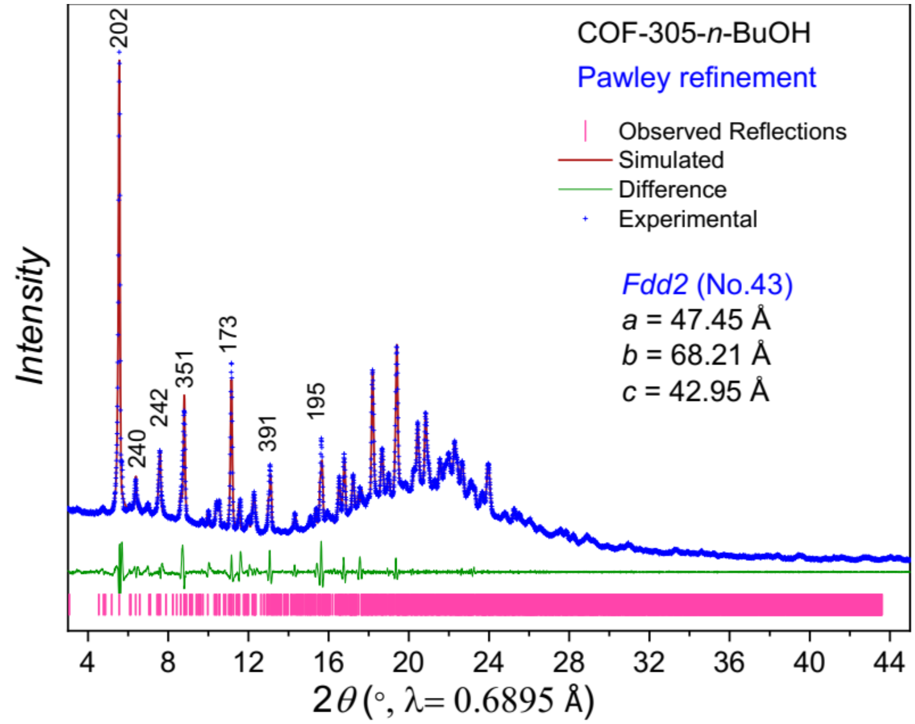

Supplementary Fig. 7. Pawley refinement of refined single crystal structure of COF-305 (red) against the PXRD pattern of the *n*-butanol adsorbed sample (blue). The experimental PXRD pattern was collected with the crystal size of ca. 40 μm.

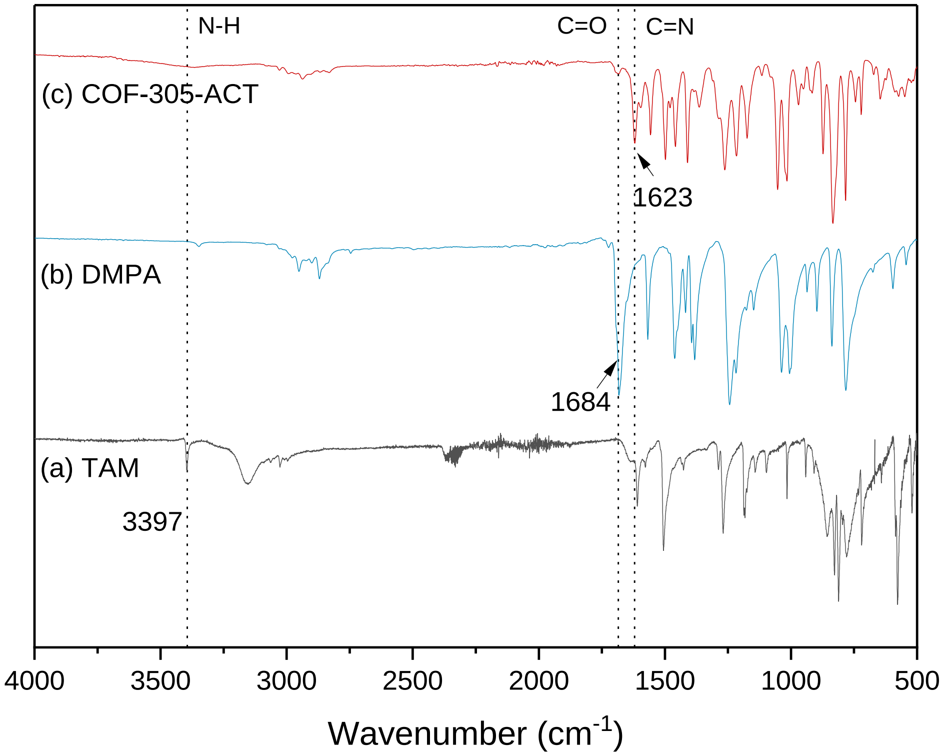

Supplementary Fig. 8. FTIR spectra of starting material TAM (a), DMPA (b) and COF-305 (c). IR peak at 3397 cm^{-1} for TAM (assigned to the N-H stretching mode of amino group) and 1684 cm^{-1} for DMPA (assigned to the C=O stretching mode of aldehyde) disappeared in COF-305. Newly emerged IR peak at 1623 cm^{-1} for COF-305 confirms the formation of imine linkage.

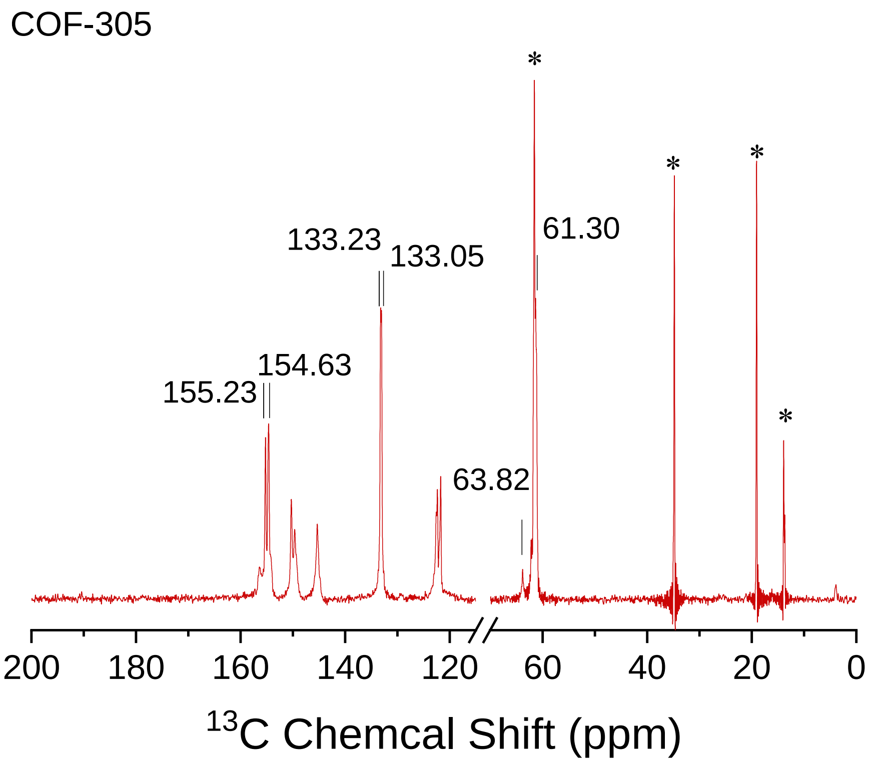

Supplementary Fig. 9. The ^{13}C SSNMR spectra of COF-305 under *n*-butanol vapor atmosphere showing the successful formation of imine linkage. The signal at 155.23 and 154.63 ppm were assigned to the carbon atom of imine bond for COF-305. The characteristic chemical shift at 61.53 ppm should be assigned to the carbon atom of the methoxy group of DMPA, and chemical shift at 63.82 ppm is assigned to the quaternary carbon atom in TAM segment. Chemical shifts at 61.569, 34.80, 19.07, and 13.90 ppm are assigned to the carbon atoms of *n*-butanol.

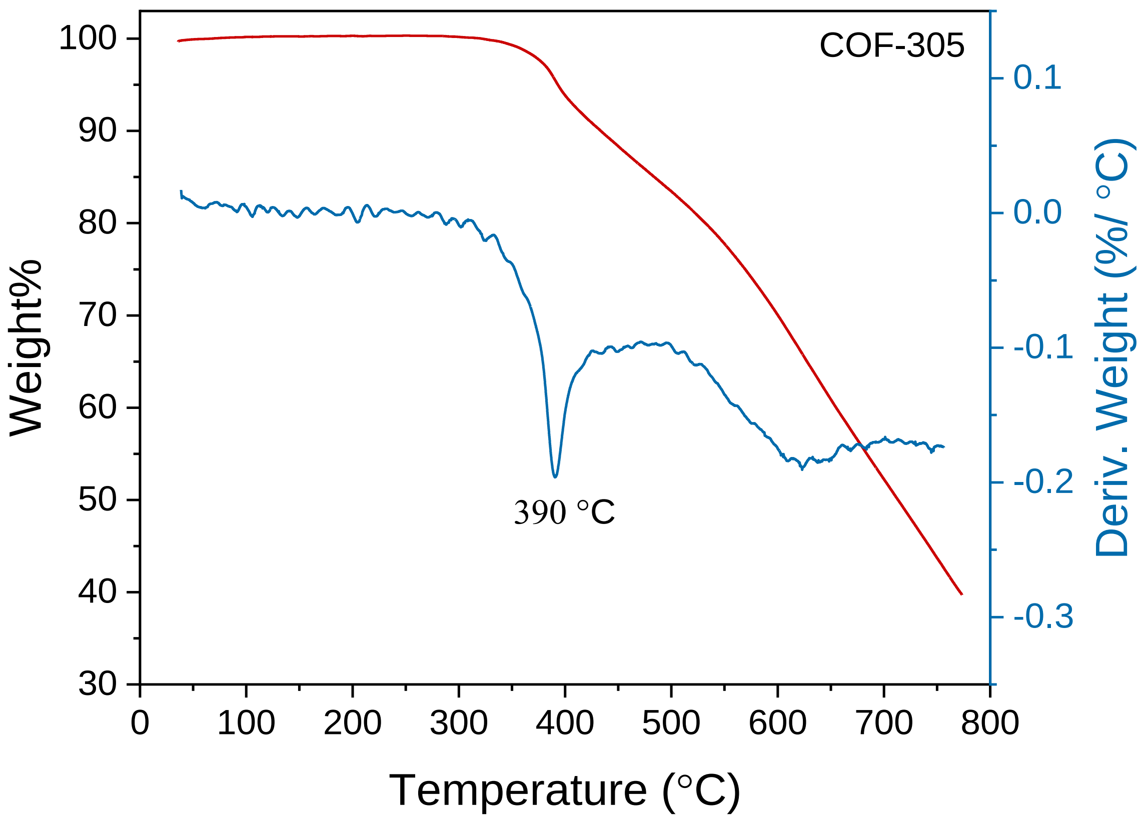

Supplementary Fig. 10. The TGA traces of activated COF-305 with thermal stability up to 390 °C.

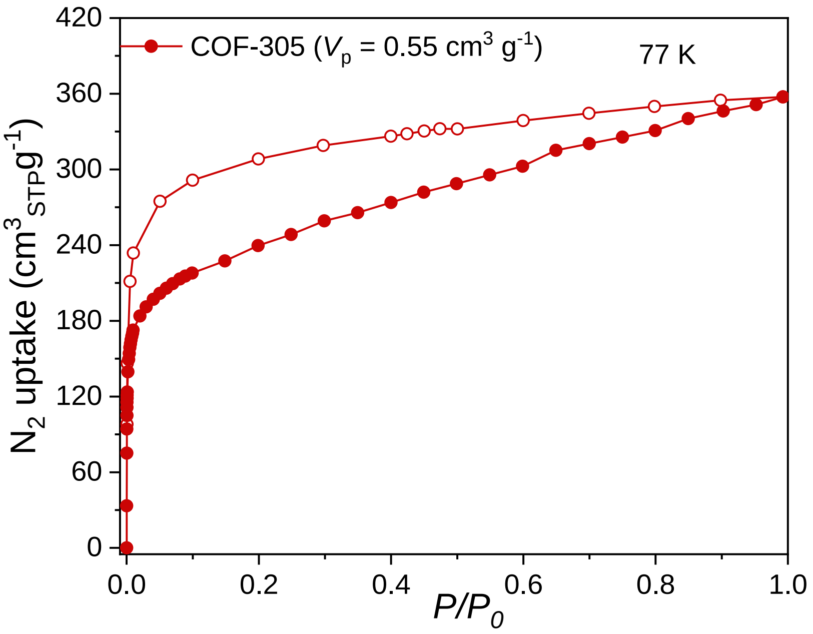

Supplementary Fig. 11. N_2 adsorption isotherm of COF-305 measured at 77 K.

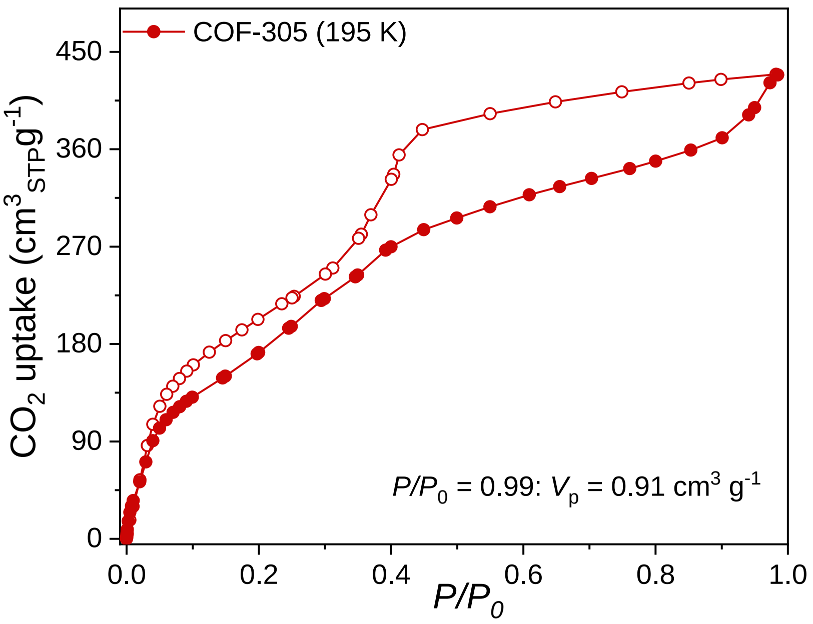

Supplementary Fig. 12. CO_2 adsorption isotherm of COF-305 measured at 195 K. CO_2 uptake shows stepwise adsorption and hysteresis between adsorption and desorption, which means the structure changes during CO_2 go through the channels.

Supplementary Table 3. Crystallographic data and structural determination for COF-304.

Name	COF-304
Formula sum	$C_{164}H_{112}N_{16}O_{16}$
Formula Weight	2562.7427
Crystal system	Tetragonal
Space group	*I*-4 (No.82)
a (Å)	26.210(6)
b (Å)	26.210(6)
c (Å)	7.582(3)
V (Å^3)	5209 (3)
Z	16
Density(g/cm^3)	0.817
Measured reflections	5222
unique reflections	1879
Θ range (º)	2.38-18.19
R_1	0.1805
wR_2	0.4147
S(GOF)	1.094
Parameters	55
Restraints	204
Max/min res. Dens., (e Å^{-3})	0.331 / -0.716
Crystal size, mm^3	0.01×0.01×0.06
Radiation, Å	0.71073
Temperature (K)	100
CCDC number	2292628

$^aR_1 = \sum||Fo| - |Fc||/\sum|F_o|$; $^bwR_2 = [\sum w(F_0^2 - F_C^2)^2/\sum w(F_0^2)^2]^{1/2}$; $^cS = [\sum w(F_0^2 - F_C^2)^2/(N_{ref} - N_{par})]^{1/2}$.

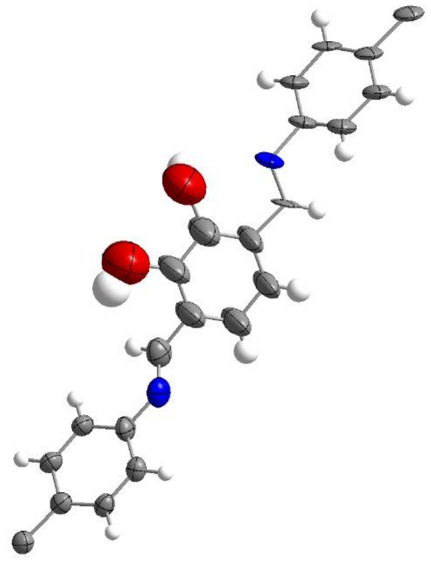

Supplementary Fig. 13. Asymmetric unit in the single-crystal structure of COF-304. Thermal ellipsoids are drawn with a 50% probability. Colour code: N, blue; O, red; C, grey; H, white.

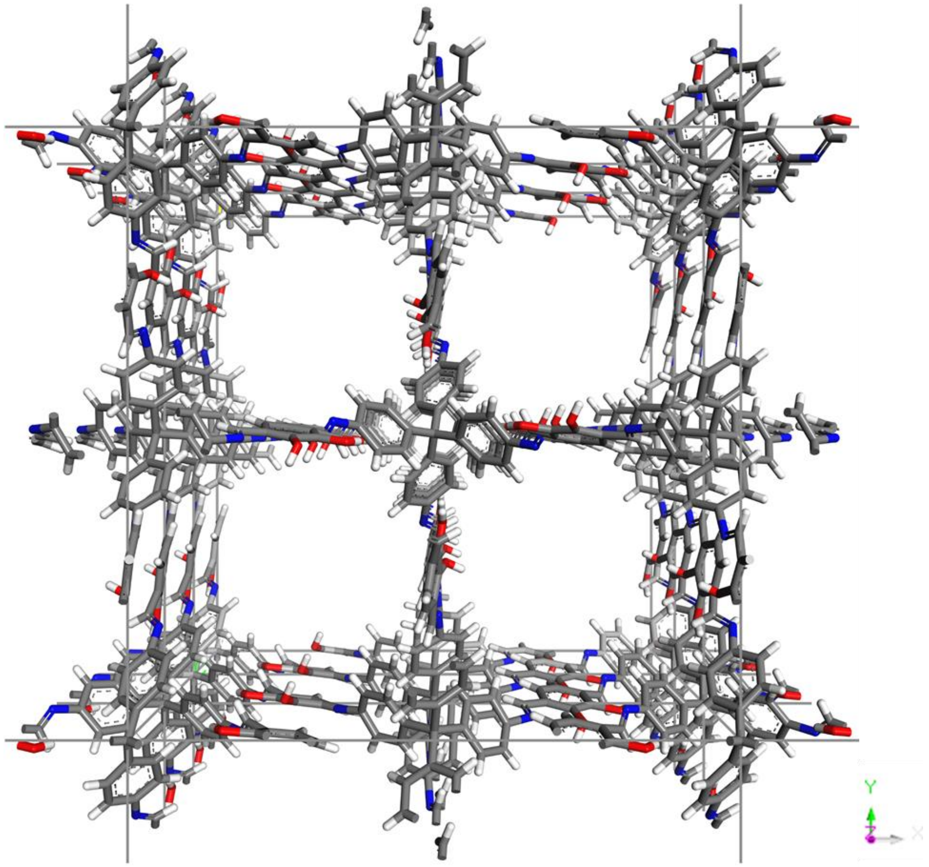

Supplementary Fig. 14. Single-crystal structure of COF-304 viewed along *c*-axis. Colour code: N, blue; O, red; C, grey; H, white.

Supplementary Note 3. Structural analysis of COF-304 and COF-305

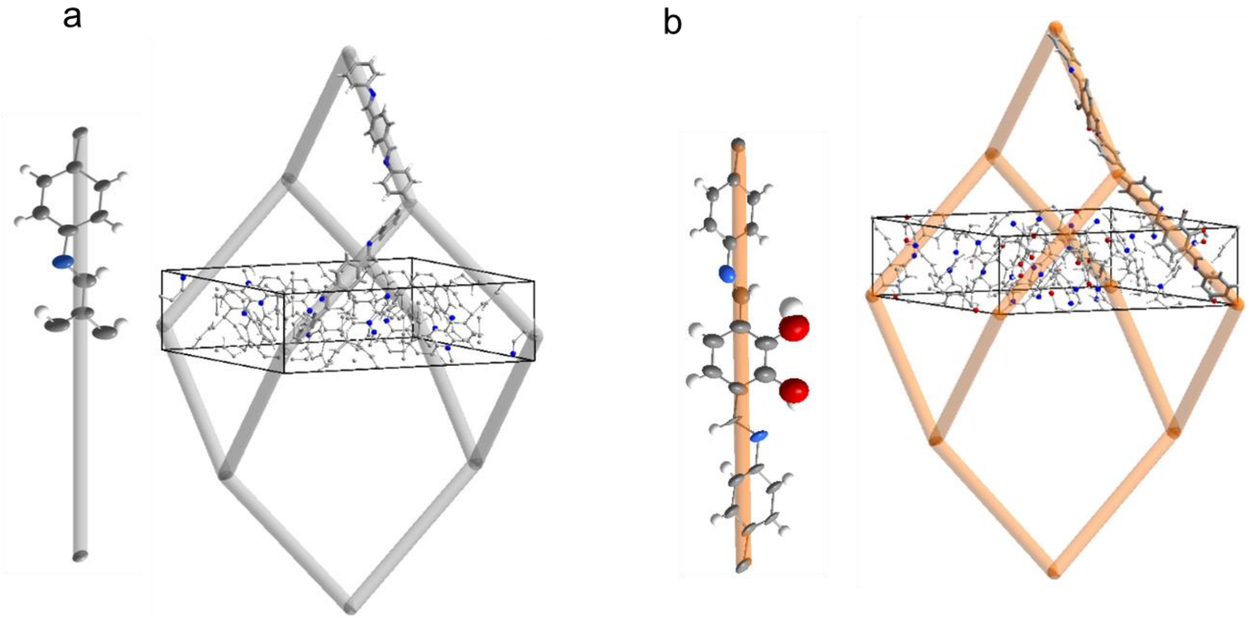

Supplementary Fig. 15. Primitive interpenetration cell (PIC) of COF-300 (a) and COF-304 (b).

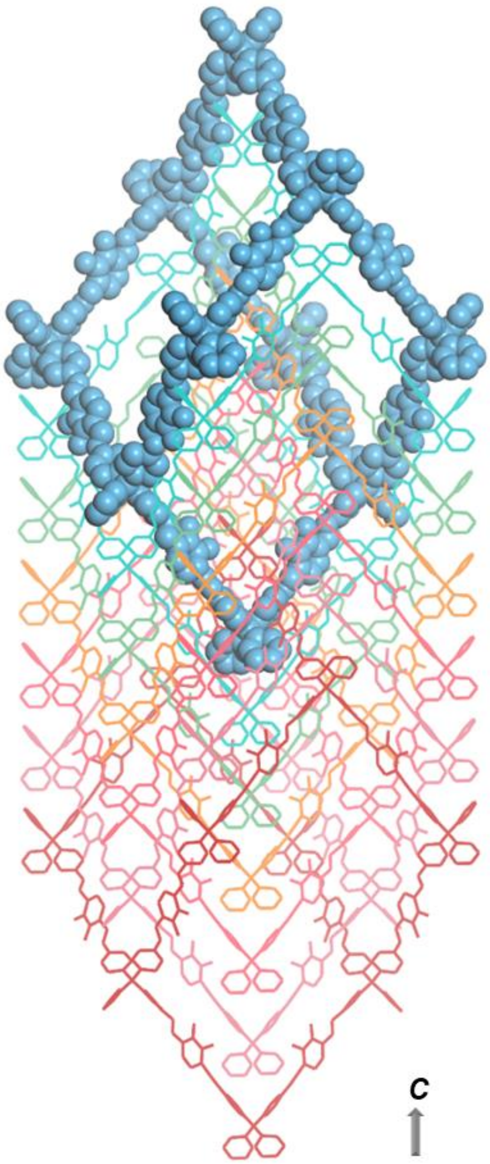

Supplementary Fig. 16. The 7-fold catenation of the diamondoid networks of COF-304.

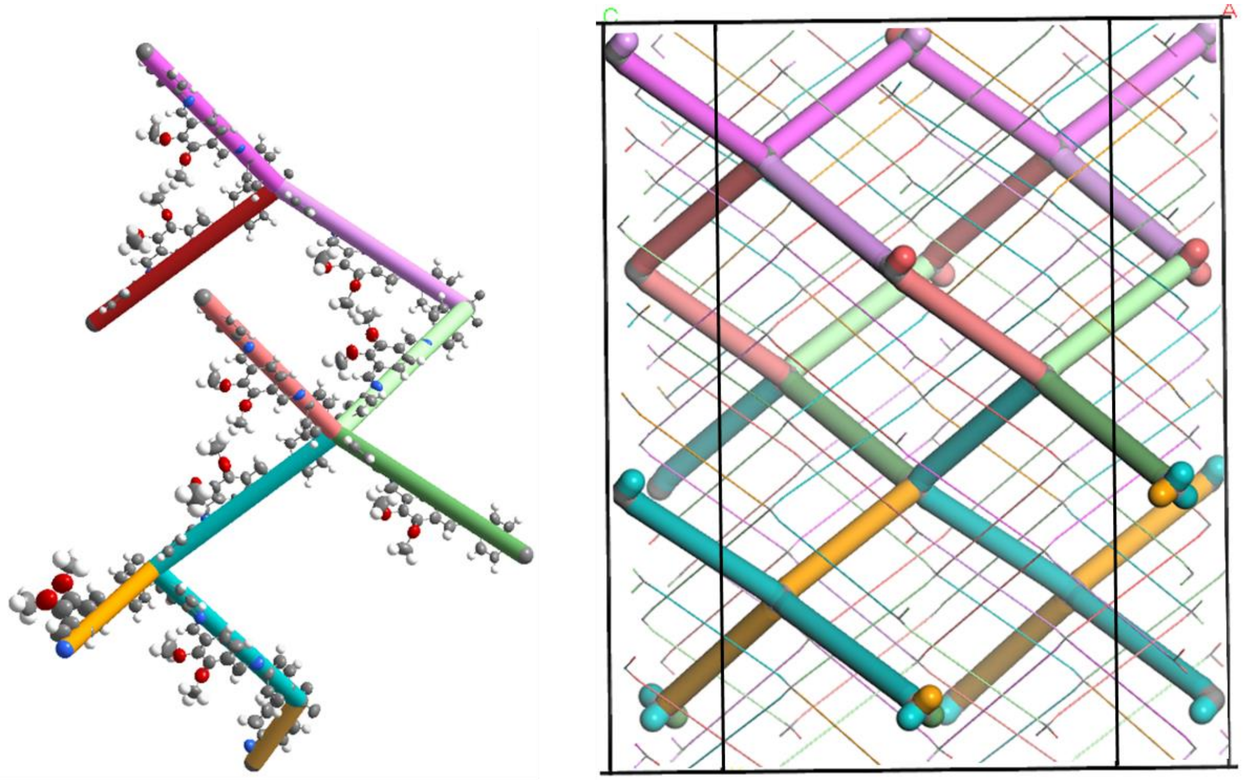

Supplementary Fig. 17. Asymmetric unit in the single-crystal structure of COF-305 and the struts are replaced by coloured sticks (left). The distribution of these sticks in the unit cell of COF-305 and we bolded one set of them (right).

Supplementary Table 4. Size of crystallographic as-symmetric unit of COFs which elucidated by *ab* initial method.

Name	topology	Unit cell	Number of as-symmetry unit
COF-904[1]	Interdigitating **hcb**	a = 25.134 Å, b = 9.659 Å, c = 18.882 Å, and β = 116.48°, V =11980.4(2) $Å^3$	1 TFB and 1.5 TMPDA
T-2DP[2]	**hcb** (ABC)	a = 26.6621(3) Å, b = 26.6621(3)Å, c = 16.8900(2) Å, γ = 120°, V =10398.0(3) $Å^3$	1/3 M1
HCP[3]	helical	a= 15.2487(11) Å, b = 19.3901(11) Å and c = 19.4093(12) Å, V =5738.5(6) $Å^3$	1 HHTP
LZU-111[4]	**lon-b-c3**	a = b = 20.17(3) Å, c = 34.0(3) Å, V =11980.4(2) $Å^3$	1 TAM and 1 TFS
Py-1P-COF[5]	AA-2D	a = 3.93 Å, b = 23.39 Å, c = 23.54 Å, α = 84.5°, β = 87.1°, γ = 87.1°, V = 2148.30 $Å^3$	2 TPA and 1 TAPy
BP-COF-6[6]		a = 5.4728(16), b = 4.3774(12), c = 14.285(4), β = 95.402(7)°, V = 340.71(16) $Å^3$	1 BPA-1
UTSB-20-qtz[7]	**qtz**	a = b = 30.935(2) Å, c = 40.760(8) Å, α = β = 90°, γ = 120°, V = 33781(8) $Å^3$	3/2 TAPB (or TFTB)
USTB-5[8]		a = b = 17.9558(6), c = 19.8313(9) Å, α = β = 90° and γ = 120°, V = 5537.2(4) $Å^3$	1/3 TAPB (or TFPB)
USTB-5r[8]		a = b = 18.2124(15), c = 17.975(2) Å, α = β = 90° and γ = 120°, V = 5163.3(10) $Å^3$	1/3 TAPB (or TFPB)
USTB-5o[8]		a = b = 31.0280(3)Å c = 39.8735(12)Å, α = β = 90° and γ = 120°, V = 33244.7(12) $Å^3$	3/4 TAPB and 3/4 TFPB
mCOF-Ag[9]	**dia**	a = 15.83 Å, b = 29.97 Å, c = 10.69 Å, and β = 123.96°, V = 4205.57 $Å^3$	1 linker
UTSB-20-dia[7]		a = 23.690(5) Å, b= 24.980(5) Å, c = 27.260(6) Å, α = 107.24(3)°, β = 107.29(3)°, γ = 106.56(2)°, V = 5209.6(10) $Å^3$	2 TAPB and 2 TFTB
COF-300[4]		a = b = 26.2260(18) Å, c = 7.5743(10) Å, V = 5209.6(10) $Å^3$	1/4 TAM and 1/2 TPA
LZU-79[4]		a = b = 27.838(2) Å, c = 7.5132(12) Å, V = 5822.4(3) $Å^3$	1/4 TAM and 1/2 BFBZ
COF-303[4]		a = b = 26.47(3) Å, c = 7.449(9) Å, V = 5220(13) $Å^3$	1/4 TFM and 1/2 PDA
COF-320[10]		a = 23.360(3) Å, c = 8.4300(17) Å, V = 4600.2(16) $Å^3$	1/4 TAM and 1/2
SYSU-9[10]		a = 26.461(4) Å, c = 7.4600(15) Å, V = 5223.6(18) $Å^3$	1/4 TAM and 1 PDD
COF-301-s[10]		a = 26.434(4) Å, c = 7.5876(15) Å, V = 5302.0(18) $Å^3$	1/4 TAM and 1/2 DHPA

COF-305		*a* = 47.077(9) Å, *b* = 67.629(14) Å, *c* = 42.547(9) Å,	4.5 TAM and 9 DMPA
COF-304		*a* = *b* = 26.210(6) Å, *c* = 7.582(3) Å	1 TAM and 1/2 DHPA

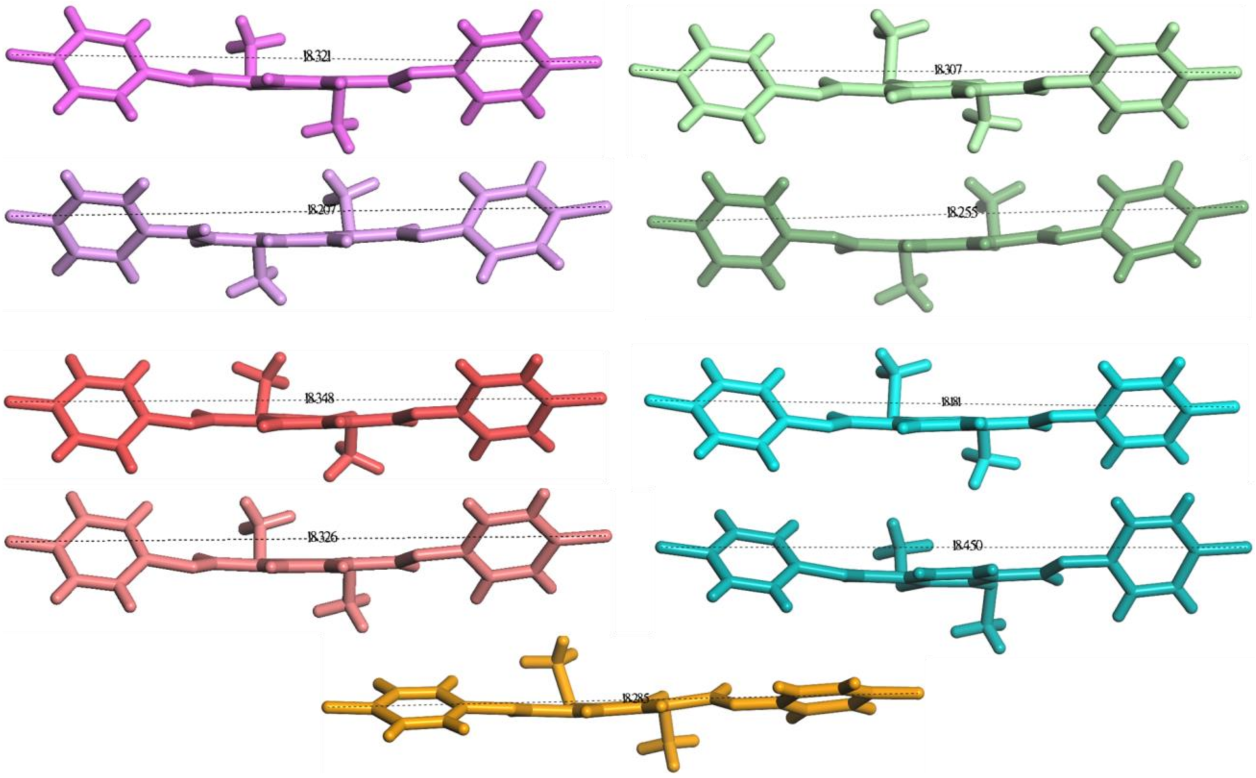

Supplementary Fig. 18. COF-305 contains 9 types of struts with varying lengths, which are measured from one carbon node to another.

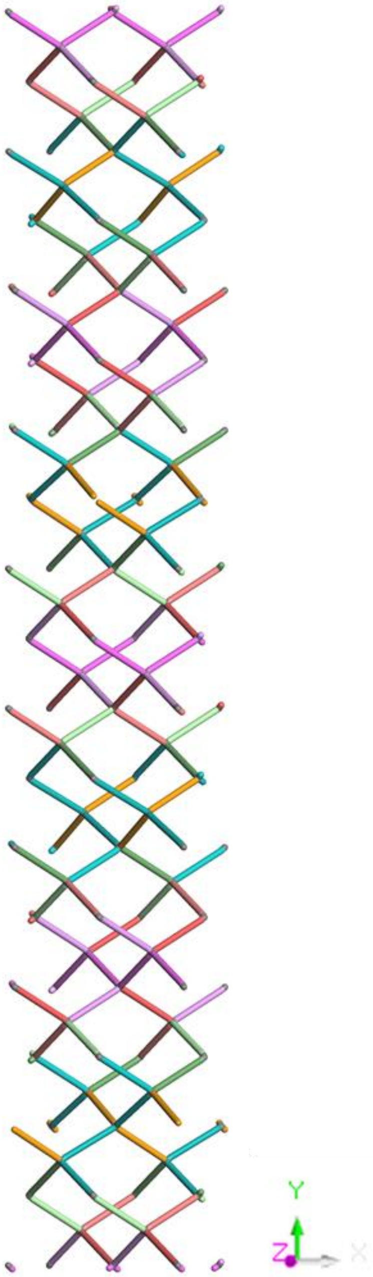

Supplementary Fig. 19. Primitive interpenetration cell (PIC) of COF-305. In one diamondoid network, the PIC is the minimum repeating unit.

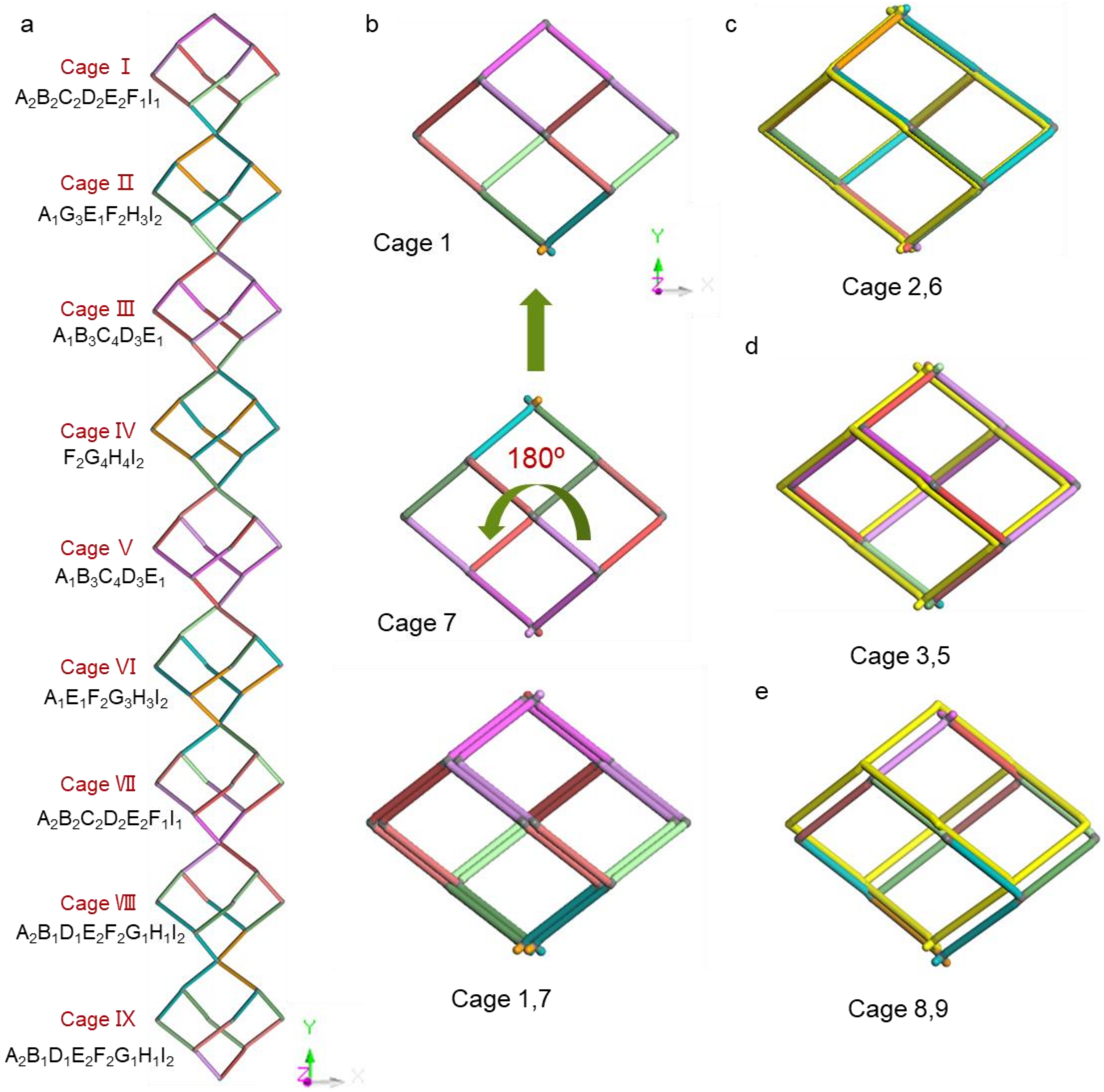

Supplementary Fig. 20. Primitive interpenetration cell (PIC) of COF-305 contains 9 kinds of cages meet at each vertex along the *b*-axis and the peripheral strut of each cage is omitted for simplicity, showing cage IV is independent (a). Of which, cage I can be obtained by symmetric operation of cage VII by rotating 180º along the *c*-axis followed by corresponding translation (b). The other three pairs of cages (2&6, 3&5, 8&9) can be obtained by similar symmetric operation (c, d, and e).

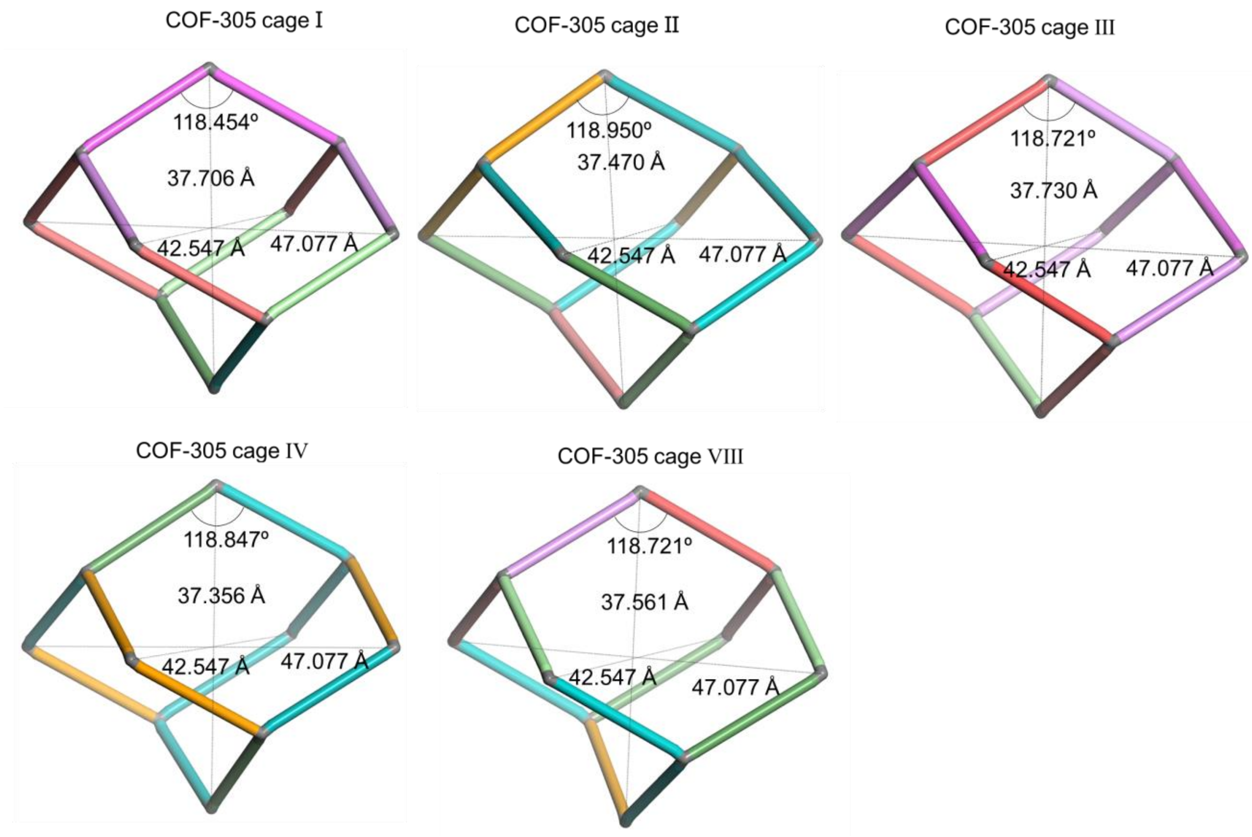

Supplementary Fig. 21. Geometry of the five non-equivalent adamantane cages of COF-305. The perpendicular distances between two vertices vary due to the difference in their composition.

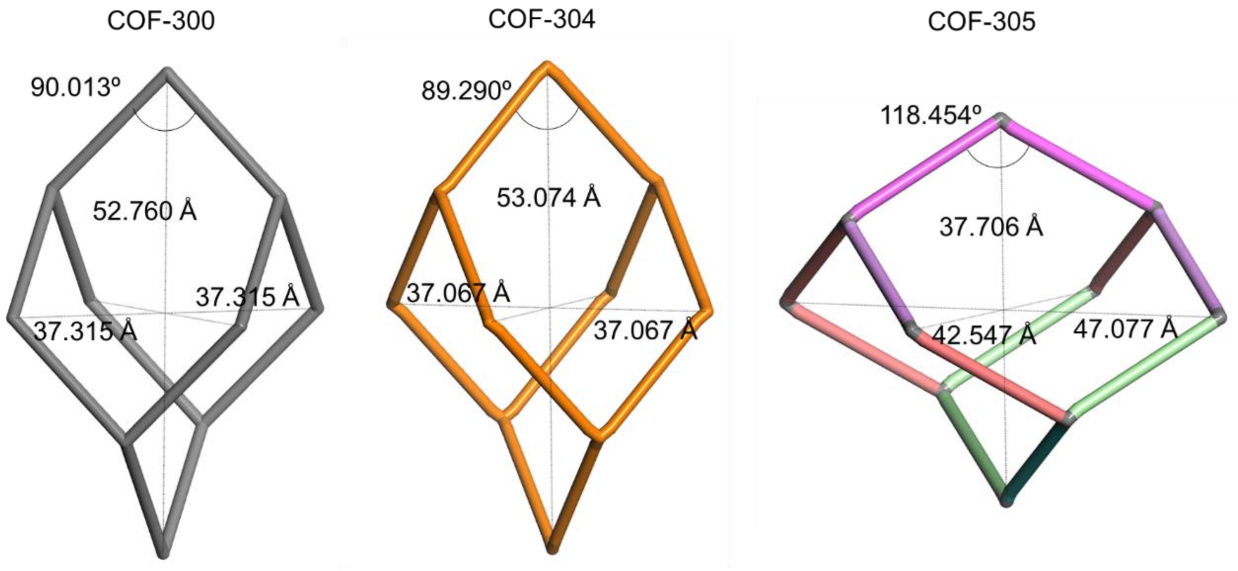

Supplementary Fig. 22. Geometry of adamantane cages of COF-300 (left), COF-304 (middle), and COF-305-cage I (right). For COF-300 and COF-304, the distance between two opposite vertices at the horizontal plane are identical, while the perpendicular distance is much larger, resulting in a deviation from the regular geometry of adamantane cage. For COF-305, there is even more deformation in the geometry of adamantane cage as the three distances are different.

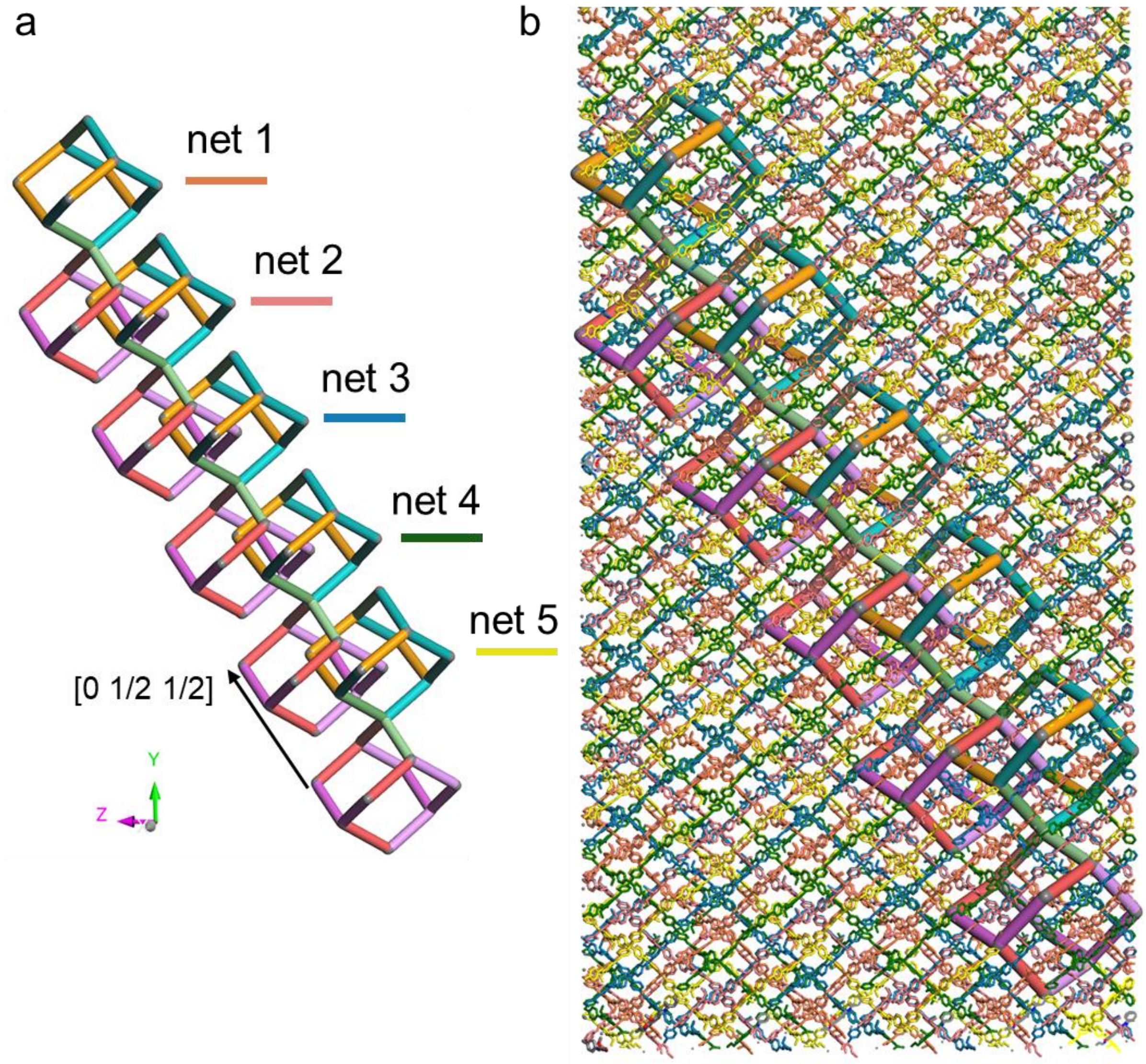

Supplementary Fig. 23. **a**, the full interpenetration vectors. A diamond net is displaced relative to each other by [0, 1/2, 1/2]. **b**, presents the position of these cages in the five nets of COF-305.

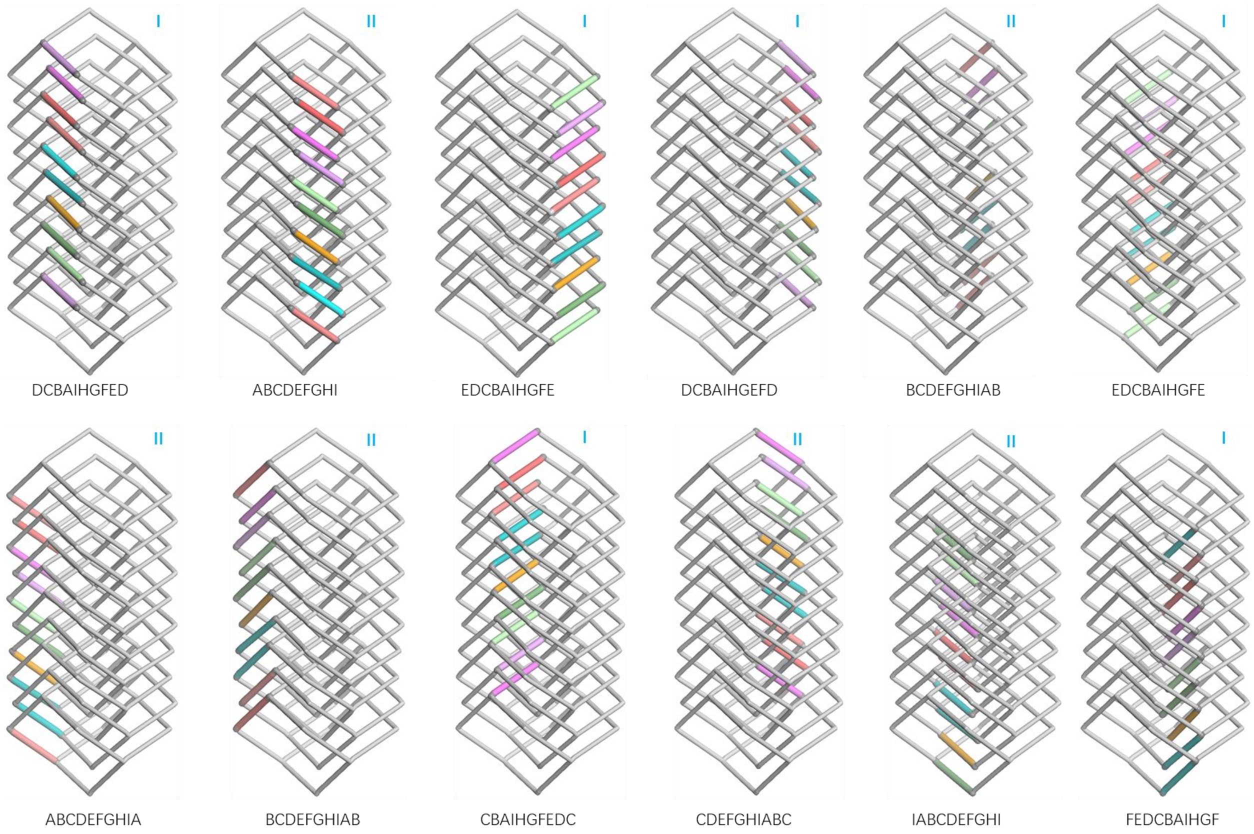

Supplementary Fig. 24. The struts arrays display two type of sequences: Type I (DCBAIHGFE) and its reverse sequence, Type II (ABCDEFGHI).

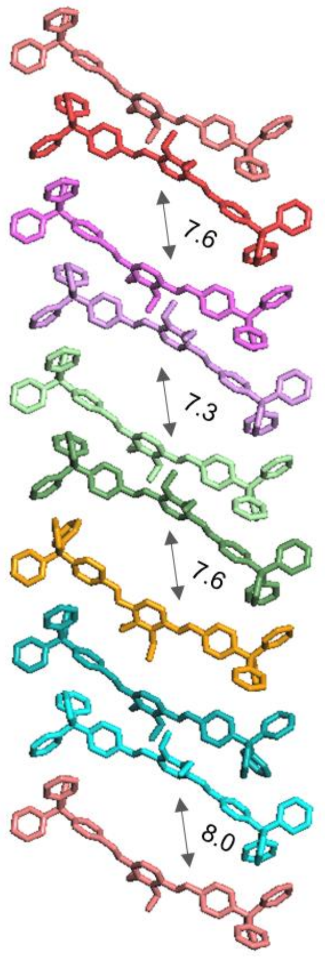

Supplementary Fig. 25. 9 types of struts arranged in a column by pairs, with the struts bent in different directions. As a result, channels are formed perpendicular to the direction of TAM stacking.

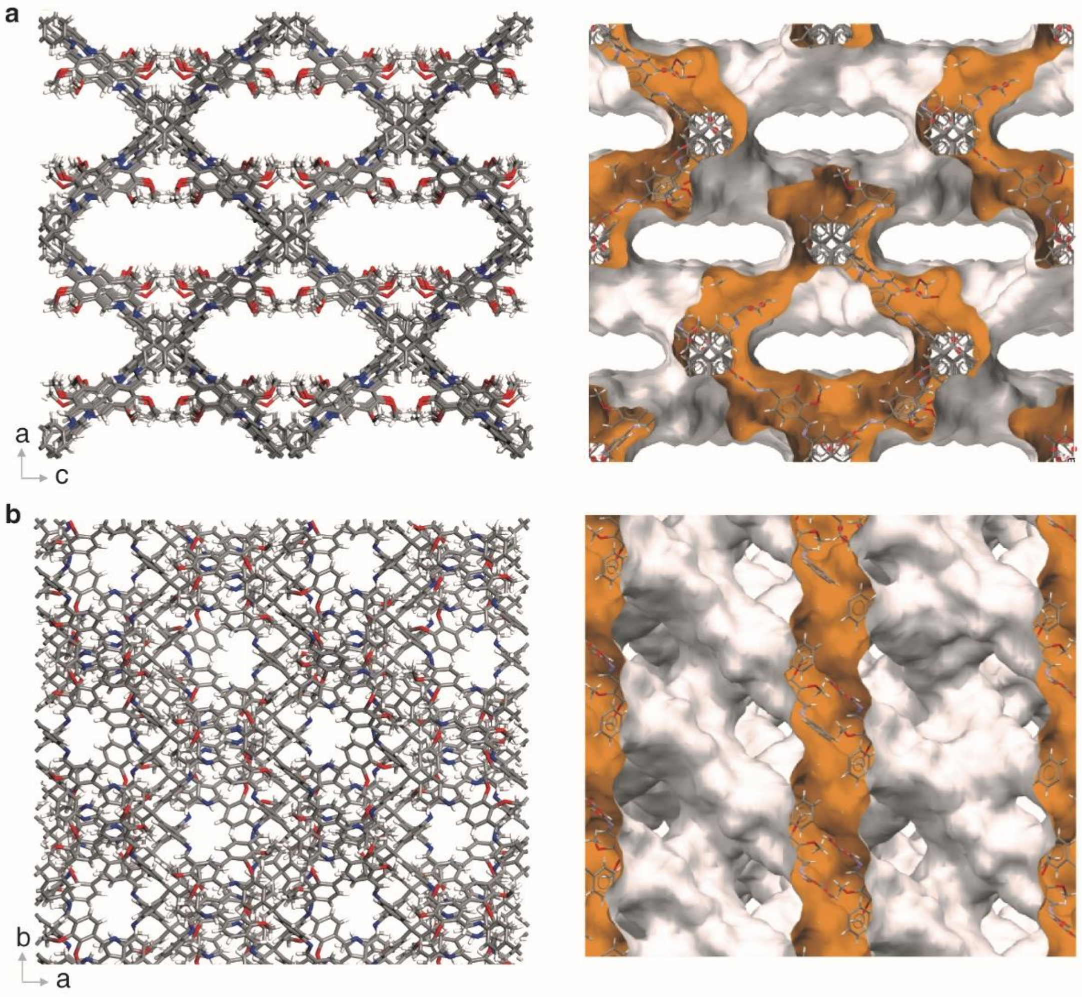

Supplementary Fig. 26. The simulated Connolly surface of COF-305 with Connolly radius of 1.0 Å, performed with Material Studio 2017 software, the simulations are based on the single-crystal structure of COF-305. a) view along *b*-axis; b) view along *c*-axis. Carbon, nitrogen, oxygen, and hydrogen atoms are in grey, blue, red, and white, respectively.

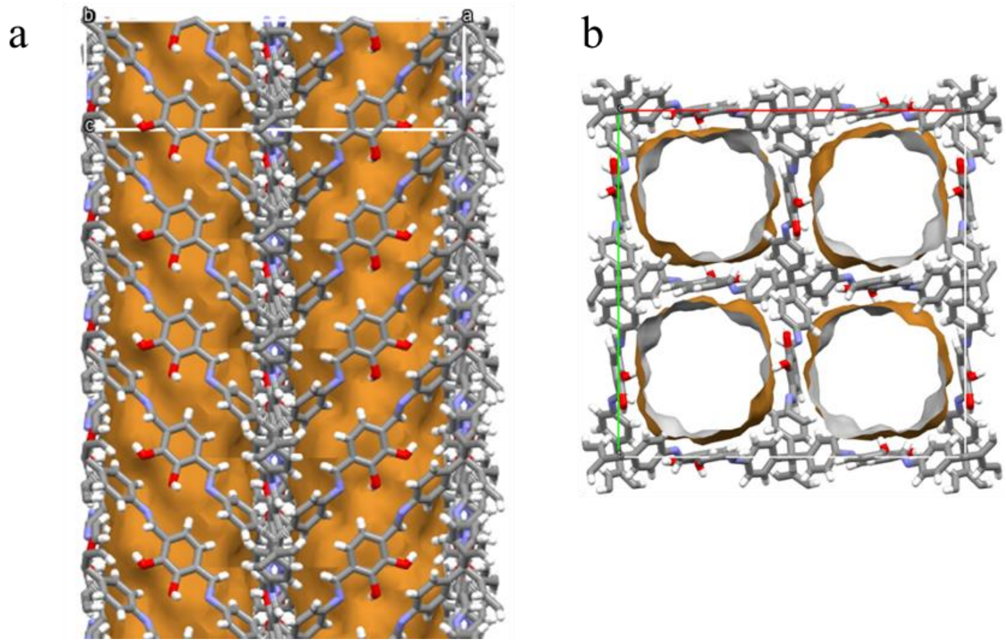

Supplementary Fig. 27. The simulated Connolly surface (orange/grey area) of COF-304 with Connolly radius of 1.0 Å performed with Material Studio 2017 software, the simulations are based on the single-crystal structure of COF-304. a) view along *a*-axis; b) view along *c*-axis. Carbon, nitrogen, oxygen, and hydrogen atoms are in grey, blue, red, and white, respectively.

Supplementary Note 4. Structure analysis of model molecules of analogue of COFs

Supplementary Table 4. Crystallographic data and structural determination for TAM.

Name	TAM
Formula sum	$C_{25}H_{24}N_4$
Formula Weight	380.4850
Crystal system	tetragonal
Space group	$I4_1/a$
a (Å)	16.7311(9)
b (Å)	16.7311(9)
c (Å)	7.1389(6)
V (Å^3)	1998.4(2)
Z	16
Density(g/cm^3)	1.265
Measured reflections	5456
unique reflections	241
Θ range (º)	4.60-31.09
R_1	0.0454
wR_2	0.1112
S(GOF)	1.0933
Parameters	72
Restraints	0
Max/min res. Dens., (e Å^{-3})	0.1107 / -0.2078
Crystal size, mm^3	0.4×0.2×0.2
Radiation, Å	1.34139
Temperature (K)	150
CCDC number	2292636

$^aR_1 = \sum||Fo| - |Fc||/\sum|F_o|$; $^bwR_2 = [\sum w\,(F_0^2 - F_C^2)^2/\sum w\,(F_0^2)^2]^{1/2}$; $^cS = [\sum w(F_0^2 - F_C^2)^2\,/(N_{ref} - N_{par})]^{1/2}$.

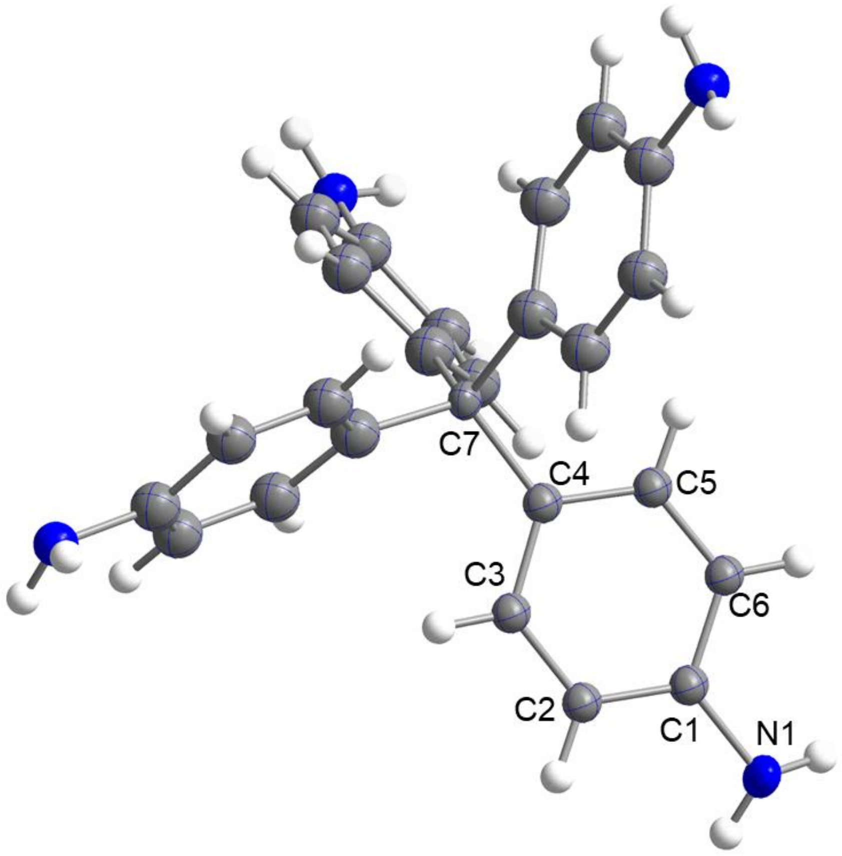

Supplementary Fig. 28. ORTEP drawing of the asymmetric unit in the crystal structure of TAM. Thermal ellipsoids are displayed with a 50% probability. Colour code: N, blue; C, grey; H, white. Symmetry-related atoms are not labeled and represented as spheres.

Supplementary Table 5. Crystallographic data and structural determination for TPM.

Name	TPM
Formula sum	$C_{50}H_{40}$
Formula Weight	640.8527
Crystal system	Tetragonal
Space group	P -4$2_1$$c$
a (Å)	10.8171(8)
b (Å)	10.8171(8)
c (Å)	7.2144(7)
V (Å^3)	844.15(12)
Z	40
Density(g/cm^3)	1.261
Measured reflections	5849
unique reflections	967
Θ range (º)	2.58-26.18
R_1	0.0396
wR_2	0.0928
S(GOF)	1.0731
Parameters	57
Restraints	0
Max/min res. Dens. ,(e Å^{-3})	0.146 / -0.186
100.0Crystal size, mm^3	0.3×0.02×0.02
Radiation, Å	0.71073
Temperature (K)	100
CCDC number	2292637

$^aR_1 = \sum||Fo| - |Fc||/\sum|F_o|$; $^bwR_2 = [\sum w\,(F_0^2 - F_C^2)^2/\sum w\,(F_0^2)^2]^{1/2}$; $^cS = [\sum w(F_0^2 - F_C^2)^2\,/(N_{ref} - N_{par})]^{1/2}$.

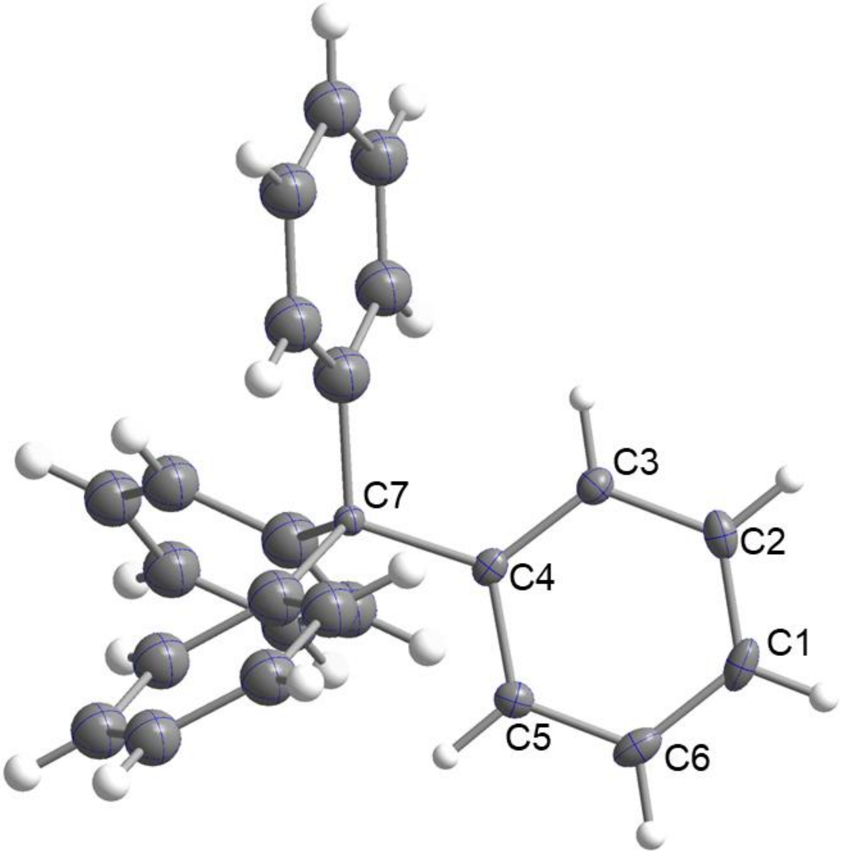

Supplementary Fig. 29. ORTEP drawing of the asymmetric unit in the crystal structure of TPM. Thermal ellipsoids are displayed with a 50% probability. Colour code: N, blue; C, grey; H, white. Symmetry-related atoms are not labeled and represented as spheres.

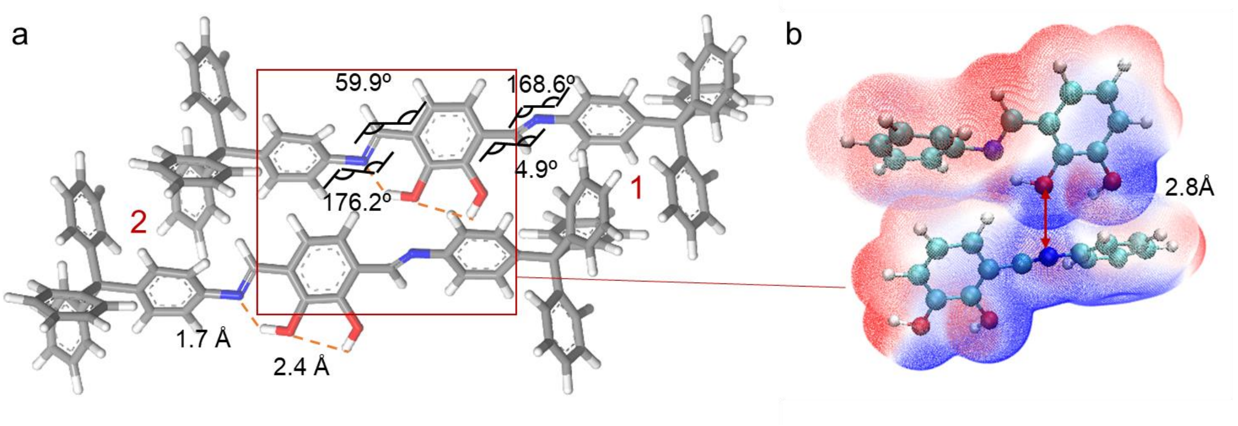

Supplementary Fig. 30. **a**. the two columns of TAM motif in COF-304 exhibits herringbone packing, albeit with different dihedral angles (dihedral angle at site 1 is 62º and at site 2 is 67º). This difference arises from the fact that one TAM has hydrogen bonding interaction with DHPA (site 2), while the other does not have any hydrogen bonding interaction (site 1). **b**, the electrostatic potential (ESP) illustrate the deviation of the dihedral angle at site 1 from 67º is attributed to steric effects that induce rotation of the imine bond, resulting in a dihedral angle of 59.9º between the imine bond and DHPA motif.

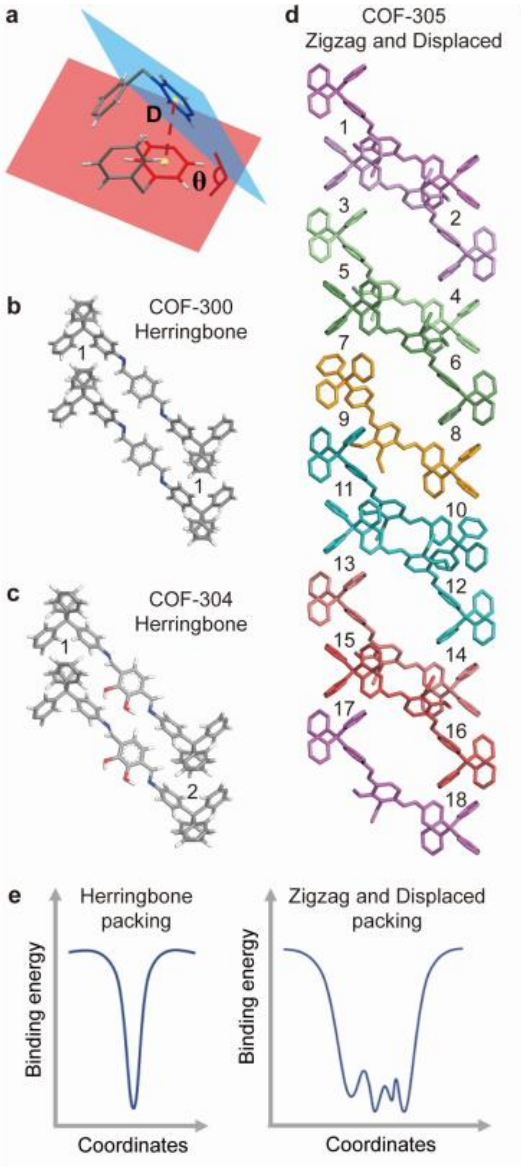

f

		Distance (Å)	Dihedral angle (θ)
COF-305	1	4.403	16.57
	2	4.395	17.35
	3	4.377	16.84
	4	4.403	16.56
	5	4.377	16.83
	6	4.453	18.77
	7	4.914/5.333	38.78/65.81
	8	4.453	19.60
	9	5.333/4.914	65.81/38.71
	10	5.333/4.914	65.81/38.78
	11	4.453	19.60
	12	4.914/5.333	38.78/65.81
	13	4.377	16.84
	14	4.453	18.77
	15	4.403	16.57
	16	4.377	16.84
	17	4.395	16.91
	18	4.403	16.57
COF-304	1	4.928	62.43
	2	5.230	66.99
COF-300		5.029	66.93
TAM		4.881	67.72
TPM		4.893	67.40

Supplementary Fig. 31. The diverse packing geometry in COF-305. a, inter-molecular interaction of TAM motif relies on two diphenylmethyl moisties and is characterized by the distance (D) and dihydral angle (θ). **b-d**, the number represent the inter-molecular interaction sites in these three COFs. **e**, the energy landscapes of TAM in crystalline state are speculated to provide rationalization for COF-305. Herringbone packing would represent one energy minimum and a high-energy barrier, so that slight deviation from the packing would result in a very unfavorable structure. When herringbone packing is not feasible for COF, the TAM go for the zigzag and displaced packing modes, which could possess multiple local energy minima with low-energy barriers, resulting in a complex structure for COF-305. **f**, table showing the distances and dihedral angles between the interacting phenyl rings of two adjacent tetraphenyl methane in COF-305, COF-304, COF-300, TAM and TPM. Numbers in the table represent inter-molecular interaction shown in the figure in the left column.

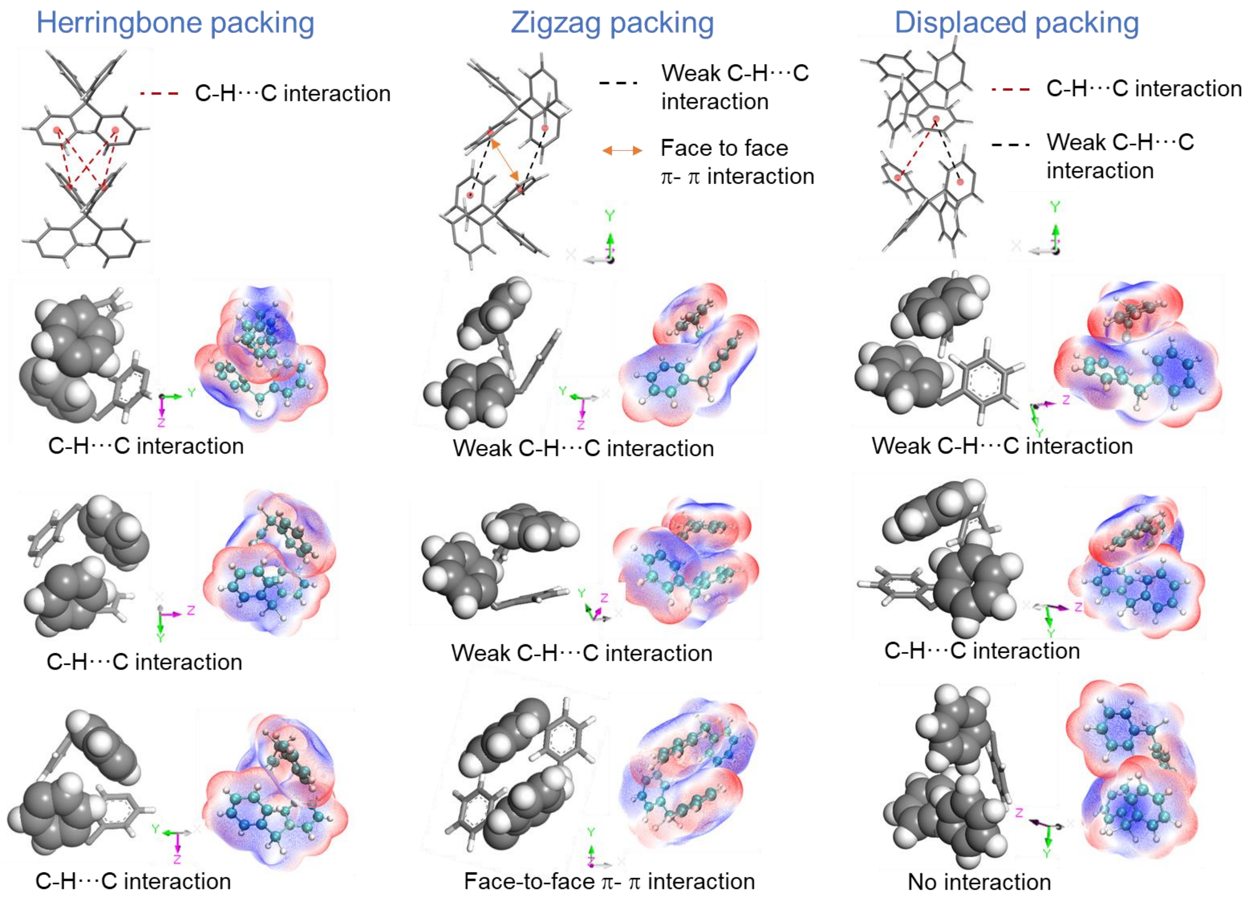

Supplementary Fig. 32. Inter-molecular interaction of TAM motif in the three packing modes. Electrostatic potential coloured van der Waals surface maps of inter-molecular interaction of TAM motif were created and the analysed phenyls pairs are in CPK mode. The TAM molecules crystallized in a highly symmetric space group, thus resulting in the four identical CH···C interactions between adjacent phenyls groups (herringbone packing). In zigzag packing mode, there exists a pair of face-to-face π-π interaction and two pairs of weak CH···C interaction. In displaced packing mode, there exists a pair of CH···C interaction and a pair of weak CH···C interaction.

Supplementary Table 6. Crystallographic data and structural determination for DHPA.

Name	DHPA
Formula sum	$C_{16}H_{12}O_8$
Formula Weight	332.2617
Crystal system	Monoclinic
Space group	$P2_1/c$
a (Å)	6.9423(2)
b (Å)	12.8713(5)
c (Å)	16.2292(5)
β (°)	95.985(2)
V (Å^3)	1442.28(8)
Z	4
Density(g/cm^3)	1.530
56	8521
unique reflections	2806
Θ range (°)	4.39 - 72.41
R_1	0.0417
wR_2	0.1495
S(GOF)	1.0400
Parameters	221
Restraints	0
Max/min res. Dens., (e Å^{-3})	-0.2976 / 0.3427
Crystal size, mm^3	0.3×0.1×0.1
Radiation, Å	1.5418
Temperature (K)	293.15
CCDC number	2292634

$^aR_1 = \sum||Fo| - |Fc||/\sum|F_o|$; $^bwR_2 = [\sum w(F_0^2 - F_C^2)^2/\sum w(F_0^2)^2]^{1/2}$; $^cS = [\sum w(F_0^2 - F_C^2)^2/(N_{ref} - N_{par})]^{1/2}$.

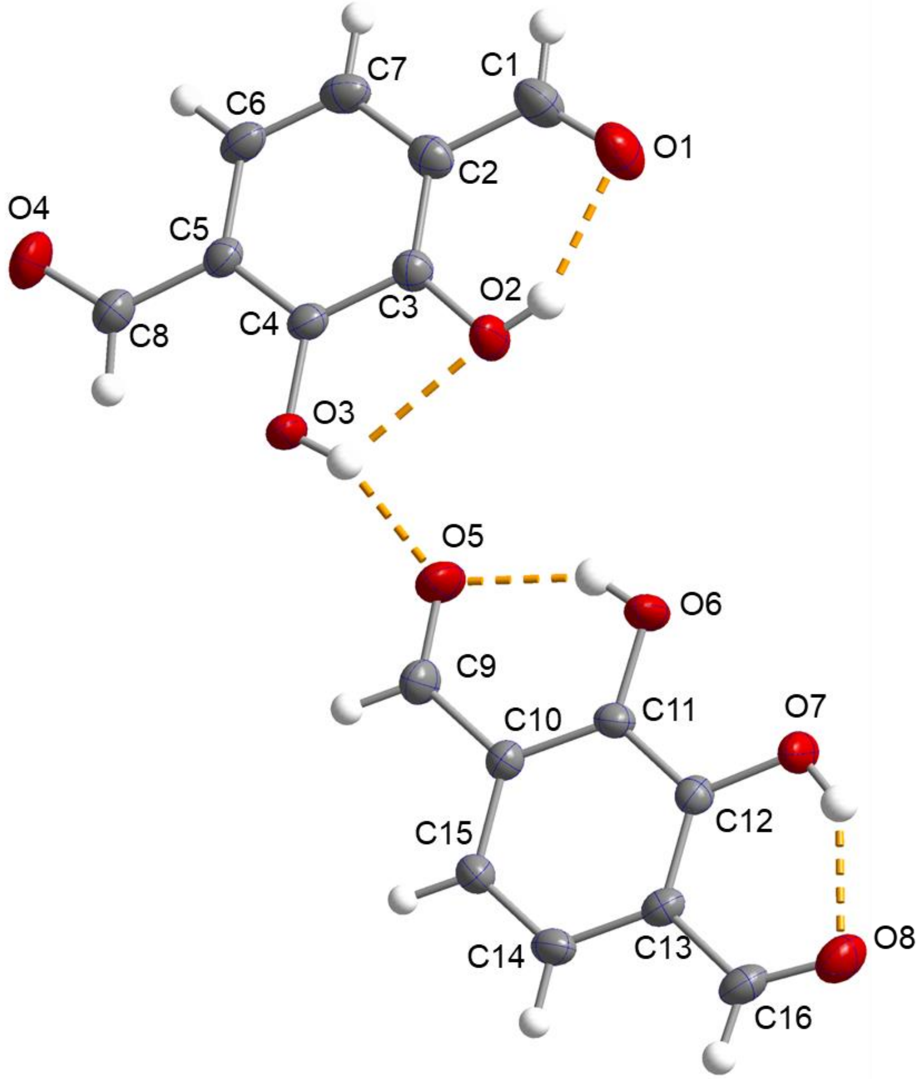

Supplementary Fig. 33. ORTEP drawing of the asymmetric unit in the crystal structure of DHPA. Thermal ellipsoids are displayed with a 50% probability. Colour code: O, red; C, grey; H, white; hydrogen bond, orange dotted line.

Supplementary Table 7. Crystallographic data and structural determination for DMPA.

Name	DMPA
Formula sum	$C_{40}H_{40}O_{16}$
Formula Weight	776.72
Crystal system	Monoclinic
Space group	$P2_1/c$
a (Å)	7.2898(7)
b (Å)	14.3216(13)
c (Å)	9.2901(9)
β (°)	108.491(5)
V (Å^3)	919.83(15)
Z	1
Density(g/cm^3)	1.402
Measured reflections	11918
unique reflections	1965
Θ range (°)	5.128 – 58.790
R_1	0.0638
wR_2	0.1717
S(GOF)	1.072
Parameters	130
Restraints	0
Max/min res. Dens., (e Å^{-3})	-0.379 / 0.276
Crystal size, mm^3	0.15×0.08×0.08
Radiation, Å	1.34138
Temperature (K)	150.0
CCDC number	2292635

$^aR_1 = \sum||Fo| - |Fc||/\sum|F_o|$; $^bwR_2 = [\sum w\,(F_0^2 - F_C^2)^2/\sum w\,(F_0^2)^2]^{1/2}$; $^cS = [\sum w(F_0^2 - F_C^2)^2/(N_{ref} - N_{par})]^{1/2}$.

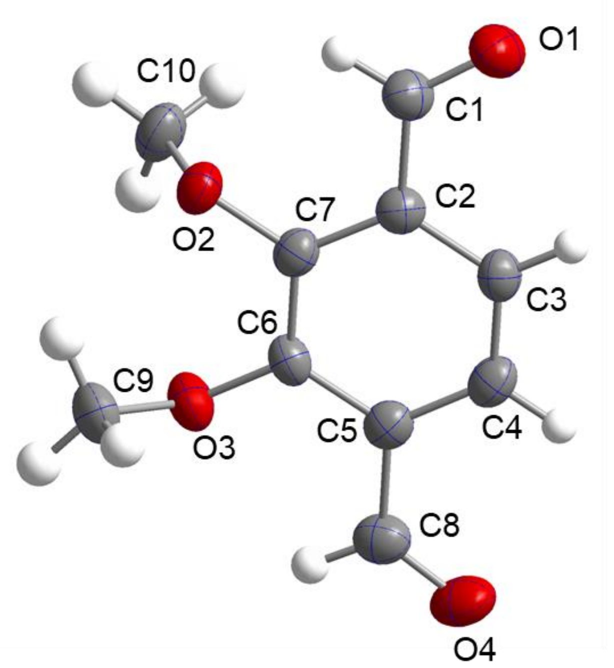

Supplementary Fig. 34. ORTEP drawing of the asymmetrical unit in the crystal structure of DMPA. Thermal ellipsoids are displayed with a 50% probability. Colour code: O, red; C, grey; H, white.

Supplementary Table 8. Crystallographic data and structural determination for M-300.

**Name**	**M-300**
Formula sum	$C_{40}H_{32}N_4$
Formula Weight	568.7090
Crystal system	Monoclinic
Space group	$P2_1/c$
a (Å)	7.4683(4)
b (Å)	6.0517(3)
c (Å)	16.3929(8)
β (°)	90.127 (2)
V (Å^3)	740.89(7)
Z	4
Density(g/cm^3)	1.275
Measured reflections	9496
unique reflections	1030
θ range (°)	4.694-47.450
R_1	0.0353
wR_2	0.0870
S(GOF)	1.052
Parameters	128
Restraints	0
Max/min res. Dens., (e Å^{-3})	-0.226 / 0.110
Crystal size, mm^3	0.3×0.1×0.1
Radiation, Å	1.34138
Temperature (K)	150
CCDC number	2292630

$^aR_1 = \sum||Fo| - |Fc||/\sum|F_o|$; $^bwR_2 = [\sum w\,(F_0^2 - F_C^2)^2/\sum w\,(F_0^2)^2]^{1/2}$; $^cS = [\sum w(F_0^2 - F_C^2)^2\,/(N_{ref} - N_{par})]^{1/2}$.

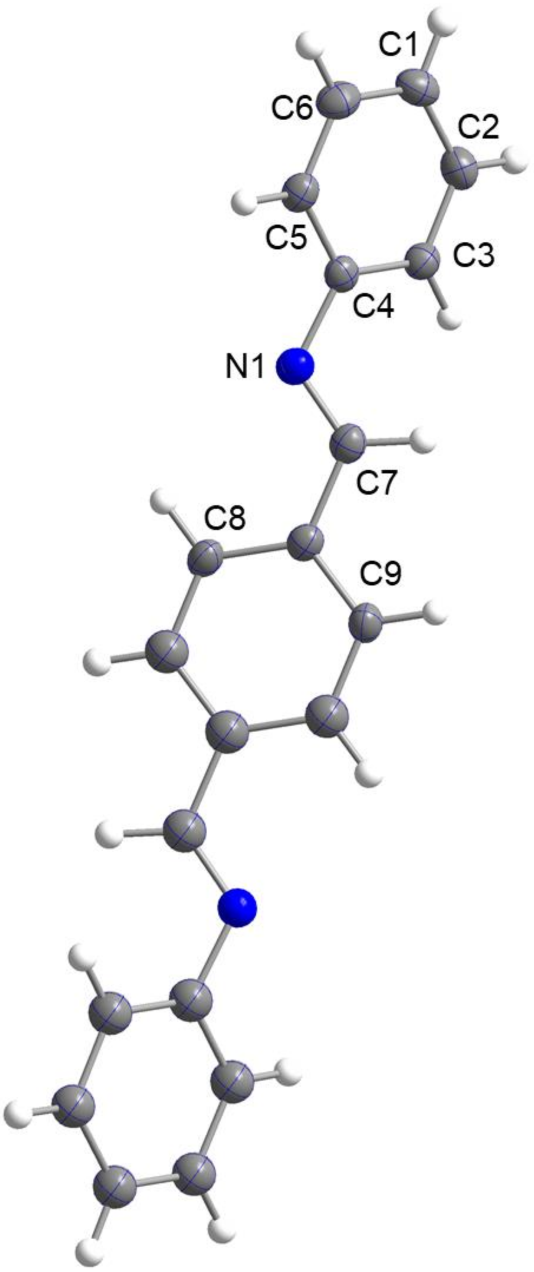

Supplementary Fig. 35. ORTEP drawing of the asymmetric unit in the crystal structure of M-300. Thermal ellipsoids are displayed with a 50% probability. Colour code: N, blue; C, grey; H, white. Symmetry-related atoms are not labeled and represented as spheres.

Supplementary Table 9. Crystallographic data and structural determination for M-304.

Name	M-304
Formula sum	$C_{80}H_{56}N_8O_8$
Formula Weight	1273.4769
Crystal system	Tetragonal
Space group	$P4_32_12$
a (Å)	6.9866(2)
b (Å)	6.9866(2)
c (Å)	32.7040 (18)
V (Å^3)	1596.37(13)
Z	8
Density(g/cm^3)	1.316
Measured reflections	12248
unique reflections	1044
Θ range (º)	4.705-46.223
R_1	0.0379
wR_2	0.0879
S(GOF)	1.077
Parameters	110
Restraints	0
Max/min res. Dens., (e Å^{-3})	-0.306 / 0.133
Crystal size, mm^3	0.2×0.2×0.01
Radiation, Å	1.34138
Temperature (K)	150
CCDC number	2292631

$^aR_1 = \sum||Fo| - |Fc||/\sum|F_o|$; $^bwR_2 = [\sum w\,(F_0^2 - F_C^2)^2/\sum w\,(F_0^2)^2]^{1/2}$; $^cS = [\sum w(F_0^2 - F_C^2)^2/(N_{ref} - N_{par})]^{1/2}$.

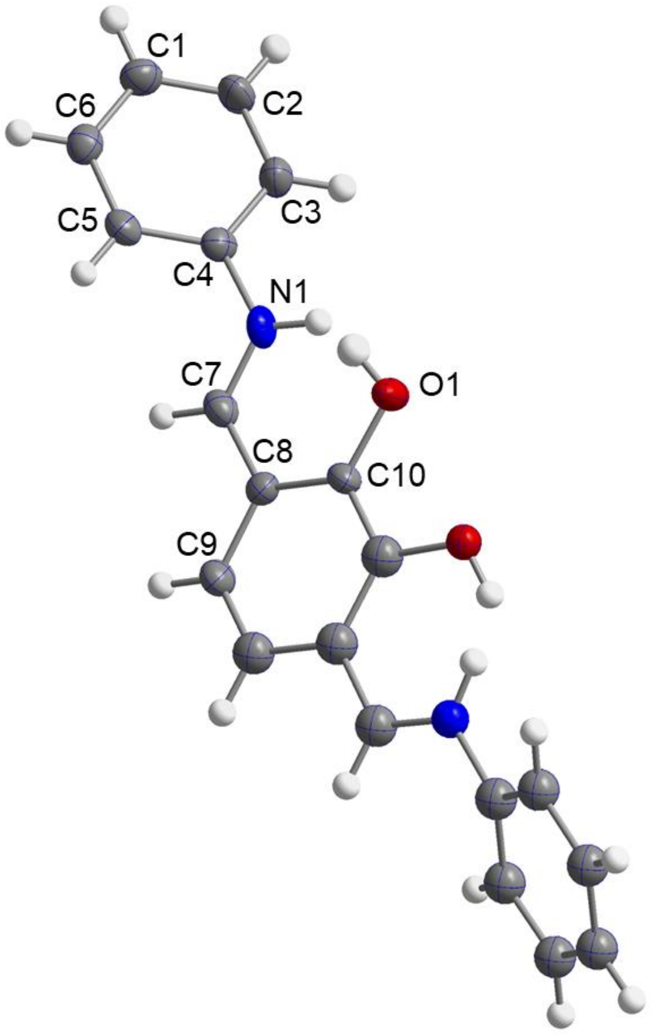

Supplementary Fig. 36. ORTEP drawing of the asymmetric unit in the crystal structure of M-304. Thermal ellipsoids are displayed with a 50% probability. Colour code: N, blue; O, red; C, grey; H, white. Symmetry-related atoms are not labeled and represented as spheres.

Supplementary Table 10. Crystallographic data and structural determination for M-305.

Name	M-305
Formula sum	$C_{88}H_{88}N_8O_8$
Formula Weight	1385.6896
Crystal system	Orthorhombic
Space group	*Pbcn* (No.60)
a (Å)	15.2464(6)
b (Å)	11.8485(5)
c (Å)	9.7805(3)
V (Å^3)	1766.82(12)
Z	1
Density(g/cm^3)	1.302
Measured reflections	16360
unique reflections	952
Θ range (°)	4.111-42.574
R_1	0.0405
wR_2	0.1064
S(GOF)	1.003
Parameters	119
Restraints	0
Max/min res. Dens., (e Å^{-3})	0.112 / -0.495
Crystal size, mm^3	0.2×0.05×0.05
Radiation, Å	1.34138
Temperature (K)	150.0
CCDC number	2292632

$^aR_1 = \sum||Fo| - |Fc||/\sum|F_o|$; $^bwR_2 = [\sum w\,(F_0^2 - F_C^2)^2/\sum w\,(F_0^2)^2]^{1/2}$; $^cS = [\sum w(F_0^2 - F_C^2)^2\,/(N_{ref}$ - $N_{par})]^{1/2}$.

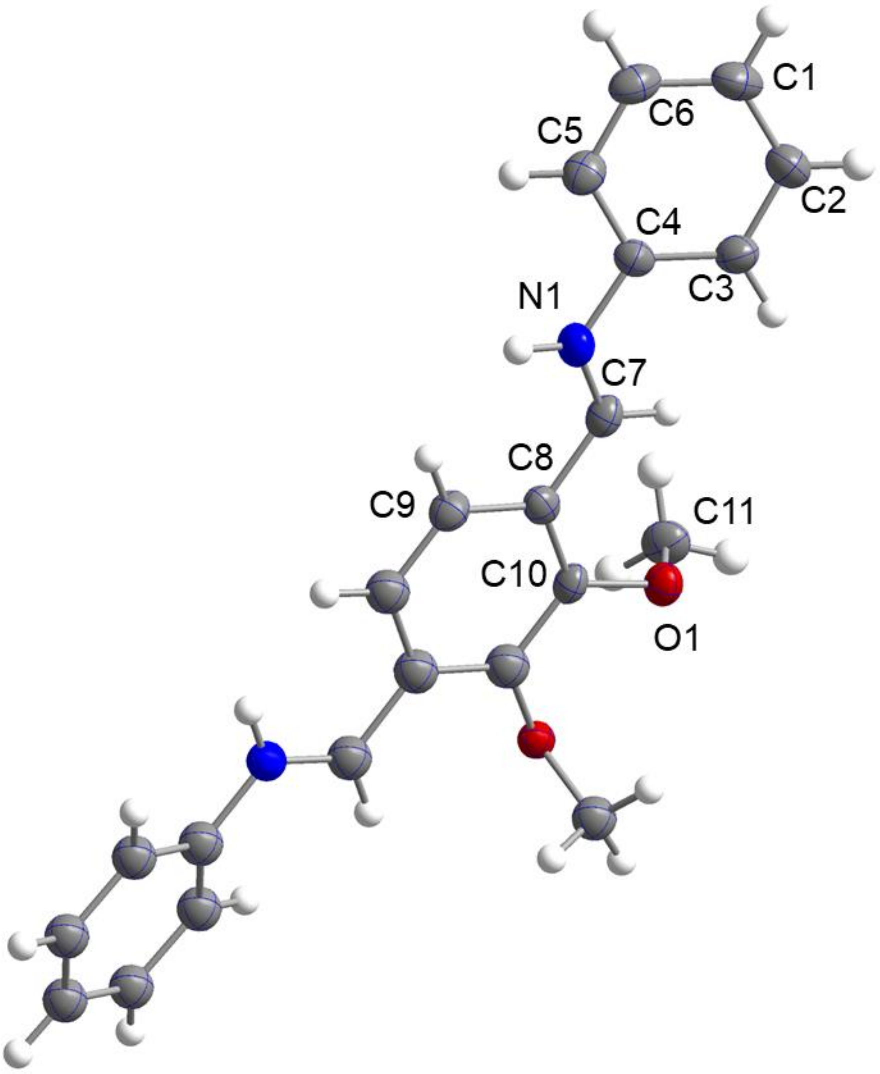

Supplementary Fig. 37. ORTEP drawing of the asymmetric unit in the crystal structure of M-305. Thermal ellipsoids are displayed with a 50% probability. Colour code: N, blue; O, red; C, grey; H, white. Symmetry-related atoms are not labeled and represented as spheres.

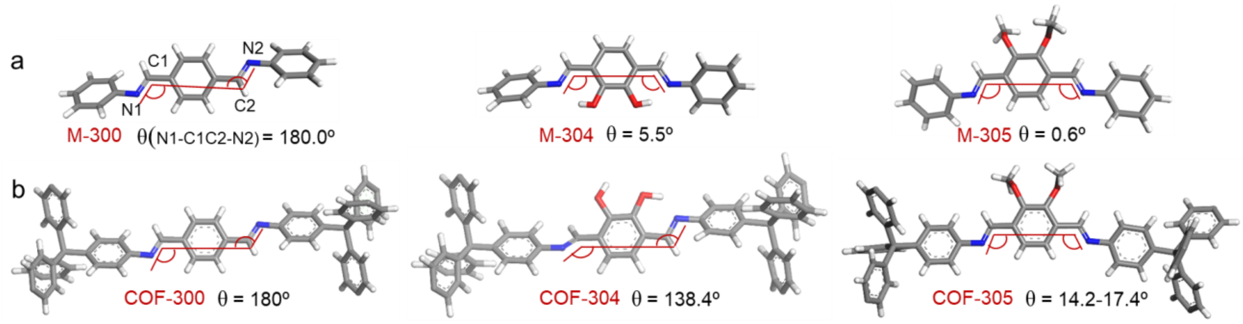

Supplementary Fig. 38. The relative configuration between the two adjacent imine bonds for model compounds (a) and COF-300 series (b). N1, C1, and C2 atoms form one plane and C1, C2, and N2 atoms form the second plane. The dihedral angle of N1-C1C2-N2 is measured. A dihedral angle (θ) close to 0º is referred to as a “C” shape, whereas an angle close to 180º is referred to as “S” shape. Consequently, COF-300, M-300, and COF-304 exhibit an “S” shape, and M-304, M-305, and COF-305 display a “C” shape.

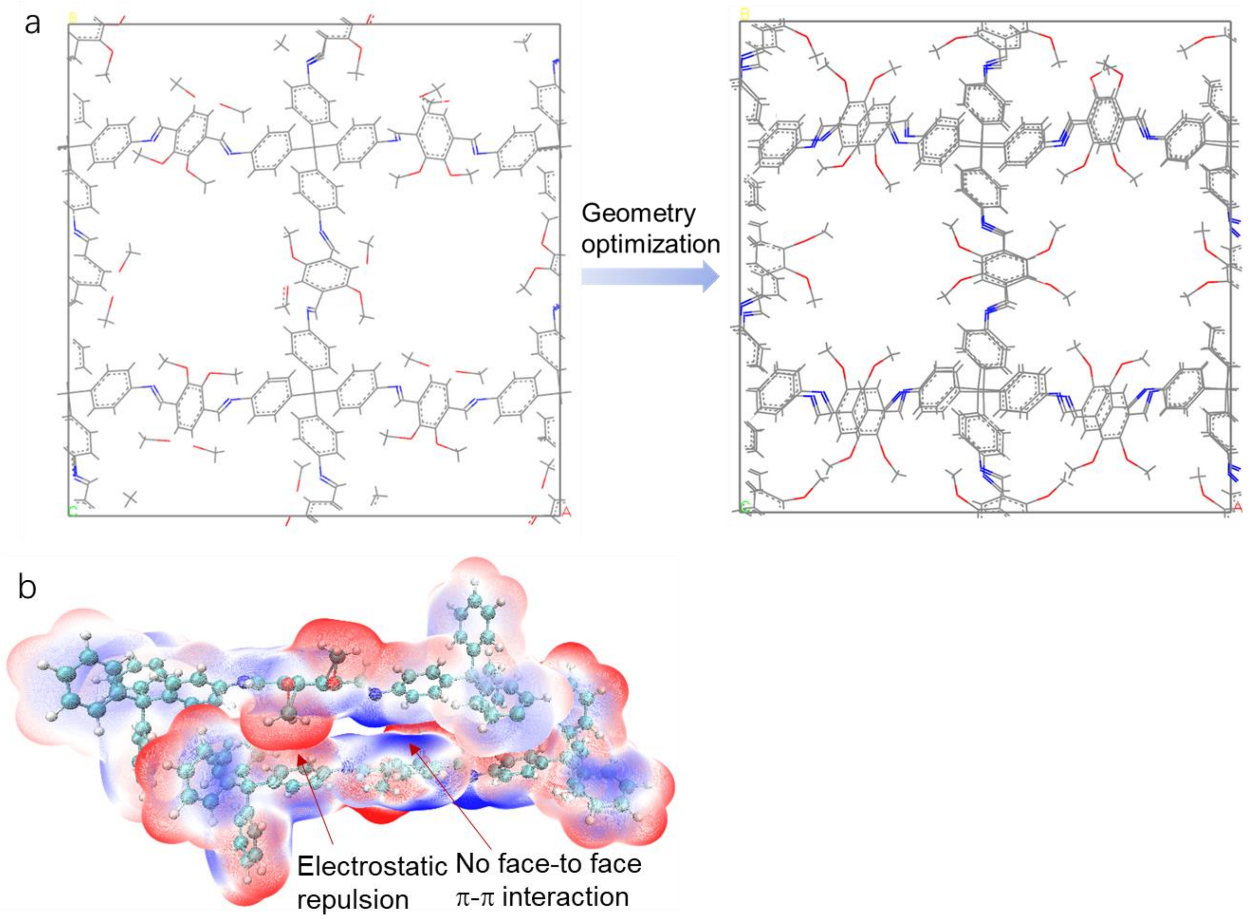

Supplementary Fig. 39. **a**, we directly stitch TAM in herringbone packing mode with DMPA in a space group of P1 with unit cell of a = 28 Å, b = 28 Å, c =15 Å. The c dimension is doubled compared to that of COF-300 due to the presence of large group of methoxy group causing significant steric hindrance in the same side. Although we put the methoxy on both sides of the phenyl ring to accommodate the adjacent DMPA, there still exists steric hindrance between the methoxy of DMPA motif and phenyl of TAM motif due to their close proximity at a distance of 1.8 Å. **b**, the electrostatic potential (ESP) illustrate the phenyl group of DMPA does not exhibit any interaction with the adjacent strut. Therefore, the COF-305 with herringbone packing similar to COF-300 could not give a feasible COF structure. And we also conducted a geometry optimization on this unfeasible structure, where the TAM packing mode offset the herringbone into a displaced mode.

Supplementary Table 11. Crystallographic data and structural determination for M-305-2.

**Name**	**M-305-2**
Formula sum	$C_{244}H_{224}N_{16}O_{32}$
Formula Weight	3892.4780
Crystal system	tetragonal
Space group	$I4_1/a$ (No.88)
a (Å)	25.5913(13)
b (Å)	25.5913(13)
c (Å)	7.7252(6)
V (Å^3)	5059.3(5)
Z	16
Density(g/cm^3)	1.283
Measured reflections	11752
unique reflections	944
Θ range (°)	3.00-36.77
R_1	0.0653
wR_2	0.1769
S(GOF)	1.0485
Parameters	167
Restraints	0
Max/min res. Dens., (e Å^{-3})	0.4916 / -0.3523
Crystal size, mm^3	0.2×0.02×0.02
Radiation, Å	1.34138
Temperature (K)	150.0
CCDC number	2292633

$^aR_1 = \sum||Fo| - |Fc||/ \sum|F_o|$; $^bwR_2 = [\sum w\ (F_0^2 - F_C^2)^2/ \sum w\ (F_0^2)^2]^{1/2}$; $^cS = [\sum w(F_0^2 - F_C^2)^2\ /(N_{ref} - N_{par})]^{1/2}$.

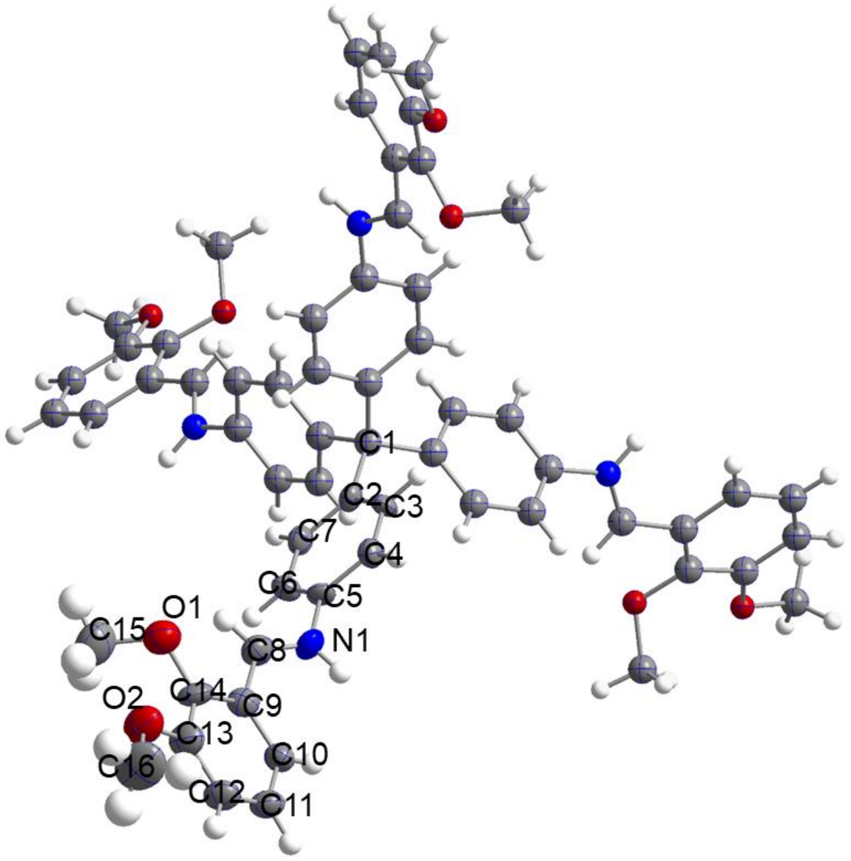

Supplementary Fig. 40. ORTEP drawing of the asymmetric unit in the crystal structure of M-305-2. Thermal ellipsoids are displayed with a 50% probability. Colour code: N, blue; O, red; C, grey; H, white. Symmetry-related atoms are not labeled and represented as spheres.

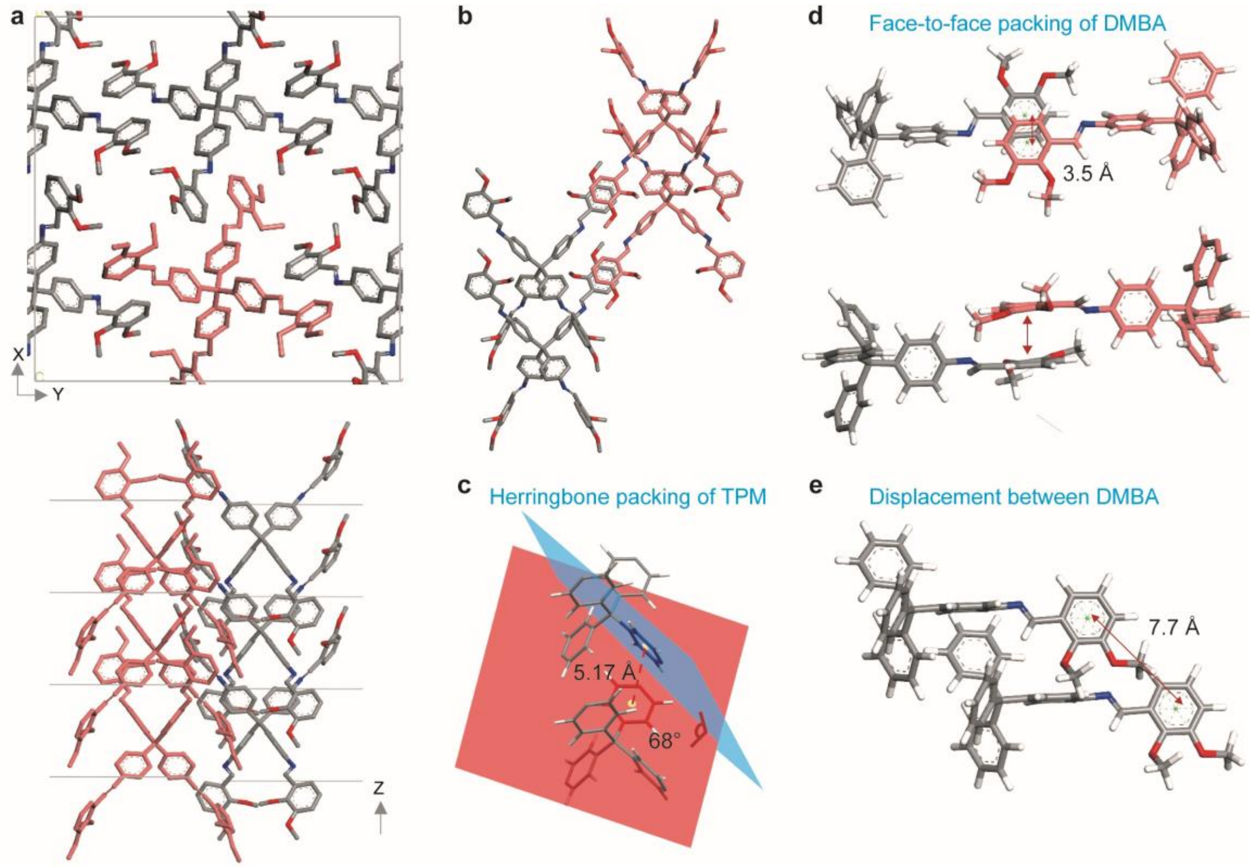

Supplementary Fig. 41. Single-crystal structure of M-305-2. a, packing of M-305-2 viewed from *c*-axis (top) and viewed along *c*-axis (down). **b,** the presentation will feature two columns of M-305-2 molecules, with one column highlighted in red. **c**, the TAM motifs in M-305-2 are arranged in herringbone packing mode, with dihedral angle of 68º and distance of 5.17 Å between the interacted phenyl rings. Adjacent DMBA motifs from two column exhibit face-to-face packing (**d**), while in the same column, they adopt displaced packing (**e**).

Supplementary Table 12. The offset of DMPA motif in COF-305, COF-304, M-305 and M-305-2.

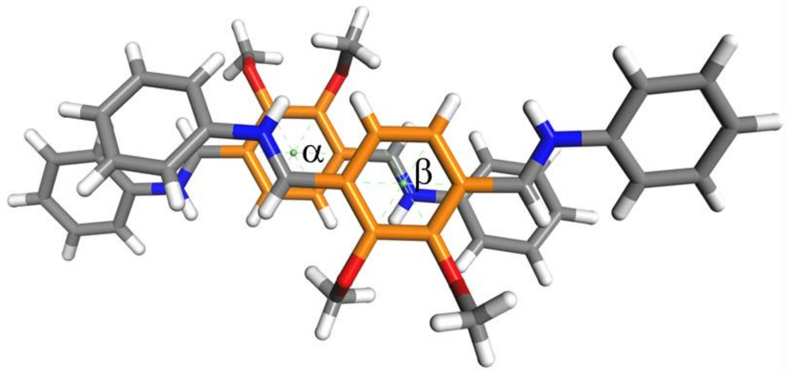

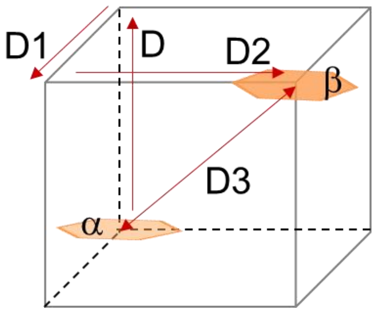

		D (Å)	D1 (Å)	D2 (Å)	D3 (Å)	Relative Energy (kJ/mol)	Absolute Energy (a.u.)
COF-305	CD	4.0	0.6	4.8	6.3	-358.7	-5152.72273555
	EF	4.0	0.1	4.8	6.3	-374.8	-5152.72888561
	HI	4.0	0.5	4.9	6.2	-176.7	-5152.65420733
	AB	4.0	0.2	4.8	6.3	-372.1	-5152.72783812
	DE	6.7	6.8	3.1	10.1	-293.5	-5152.69790319
	IA	7.4	6.1	2.2	10.0	-229.8	-5152.67367628
	BC	6.7	7.1	2.7	10.1	-262.0	-5152.68591049
	FG	6.7	6.3	2.9	10.0	-79.20	-5152.61628847
	GH	4.4	5.3	3.9	7.7	0	--5152.58612879
M-305		3.7	0.7	3.4	5.0	——	——
M-305-2		3.4	1.1	0.1	3.5	——	——

Supplementary References

1 Xiao, Y. et al. Constructing a 3D Covalent Organic Framework from 2D hcb Nets through Inclined Interpenetration. *J. Am. Chem. Soc. 145*, 13537-13541, doi:10.1021/jacs.3c03699 (2023).

2 Hu, F. et al. Highly Efficient Preparation of Single-Layer Two-Dimensional Polymer Obtained from Single-Crystal to Single-Crystal Synthesis. *J. Am. Chem. Soc. 143*, 5636−5642, doi:10.1021/jacs.1c00907 (2021).

3 Hu, Y. et al. Single crystals of mechanically entwined helical covalent polymers. *Nat. Chem. 13*, 660-665, doi:10.1038/s41557-021-00686-2 (2021).

4 Ma, T. Q. et al. Single-crystal x-ray diffraction structures of covalent organic frameworks. *Science*, *361*, 48-52, doi:10.1126/science.aat7679 (2018).

5 Kang, C. et al. Growing single crystals of two-dimensional covalent organic frameworks enabled by intermediate tracing study. *Nat. Commun. 13*, 1370, doi:10.1038/s41467-022-29086-x (2022).

6 Gropp, C., Ma, T., Hanikel, N. & Yaghi, O. M. Design of higher valency in covalent organic frameworks. *Science 370*, 424-431 doi:10.1126/science.abd6406 (2020).

7 Yu, B. et al. Observation of Interpenetrated Topology Isomerism for Covalent Organic Frameworks with Atom-Resolution Single Crystal Structures. *J. Am. Chem. Soc. 145*, 25332−25340, doi:10.1021/jacs.3c09001 (2023).

8 Yu, B. et al. Linkage conversions in single-crystalline covalent organic frameworks. Nat. Chem. 2023 doi:10.1038/s41557-023-01334-7 (2023).

9 Xu, H.-S. et al. Single crystal of a one-dimensional metallo-covalent organic framework. *Nat. Commun.* **2020**, *11*, 1434, doi:10.1038/s41467-020-15281-1

10 Zhou, Z. et al. Growth of single-crystal imine-linked covalent organic frameworks using amphiphilic amino-acid derivatives in water. *Nat. Chem. 15*, 841–847, doi:10.1038/s41557-023-01181-6 (2023).
